# Supplementary material for: Strategic Planning for Tuberculosis Control in the Republic of Fiji
Source: Trop Med Infect Dis. 2019 Apr 24;4(2):71. doi: 10.3390/tropicalmed4020071 (PMC6631049; doi:10.3390/tropicalmed4020071)
Supplement: Supplementary file 1 [file tropicalmed-04-00071-s001.pdf]

Appendix A Strategic Planning for Tuberculosis Control in the Republic of Fiji

General approach

This Supplement describes our approach to simulating the TB epidemic in Fiji. As described in our previous publication [1], we have developed a flexible modelling platform that first divides the population into compartments related to their TB-related status, specifically: susceptible, early latent, active, detected, early treatment, late treatment, and recovered groups or compartments.

The entire population is stratified according to risk groups, age groups, and treatment history groups (new and retreatment), with these stratifications applied to all the population groups and compartments, such that these stratifications are fully multiplicative. Stratification by strain and by access to care of variable quality (which is an available option in the AuTuMN platform) are not applicable to this application to Fiji. Stratification by organ manifestation (smear-positive, smear-negative, and extrapulmonary) is applied only to the compartments that represent active disease, and hence is not applied to susceptible, latently infected, and recovered groups.

Model parameters may be time-variant and informed by reported data over multiple time points or constant throughout a model run. Of the constant parameters, some are varied from run to run during the calibration process, while the remainder remain fixed throughout. This is intended to capture a high degree of historical consistency with TB epidemiology in Fiji, incorporating both TB-related and demographic aspects.

Last, our approach to calibration allows for variation in highly uncertain epidemiological quantities that are relevant to TB transmission, permitting both model calibration and quantification of the uncertainty around baseline extrapolations of the trajectory of TB burden markers. By contrast, to quantify uncertainty around the effects of interventions, we do not consider the uncertainty around disease-specific model parameters, aiming instead to isolate and quantify uncertainty relating to the evidence for the interventions alone.

Parameter types

There are four broad types of parameters implemented:

1. Constant parameters
   - These parameters are set at a constant value throughout time during the course of a model run and over sequential runs during an uncertainty simulation.
2. Calibration parameters
   - These parameters are a limited subset of the time-invariant parameters (five in this application), which are varied between consecutive runs of the model during the process of calibration and estimation of epidemiological uncertainty.
3. Time-variant parameters
   - Functions mapping parameter values to time are created by fitting to multiple data points provided for different times. This approach allows programmatic parameters to vary in order to reflect the programmatic history of Fiji and so enhance realism.
   - Polynomial spline fitting techniques are used to fit curves to available data.
4. Calculated parameters
   - Some parameters are calculated from simulated quantities at each time step of model integration. In this application, this only applies to the forces of infection for each group that is susceptible to infection. Frequency-dependent transmission and homogeneous mixing is assumed, such that the force of infection is proportional to a weighted proportion of all groups with active TB in Fiji who have not yet entered the non-infectious phase of treatment. The force of infection is modified for persons who are already infected and in late latency, for BCG-vaccinated persons, and for persons completing treatment for active disease (with the parameters reflecting partial immunity being fixed for the first two of these groups and varied with calibration for the third).

Scale-up functions

This section illustrates our fitting of the values for the third parameter type described in the previous section. For each of the following figures, the blue circles represent the data points fitted to (data sources vary), and the black line represents the parameter value used in model integration by calendar year. The left and right panels present the same data and only differ in the period of time (horizontal axis) displayed. The effective value of some of these parameter values (e.g. changes to case detection rate through active case finding) may also be modified during the analysis of specific scenarios, but only beyond 2016, with the baseline values presented here.

Bounded polynomial spline functions are used for data fitting, where the range of the function is restricted as appropriate. That is, the range of fitted functions is limited to positive values for all the functions and to values of one or less for proportions.

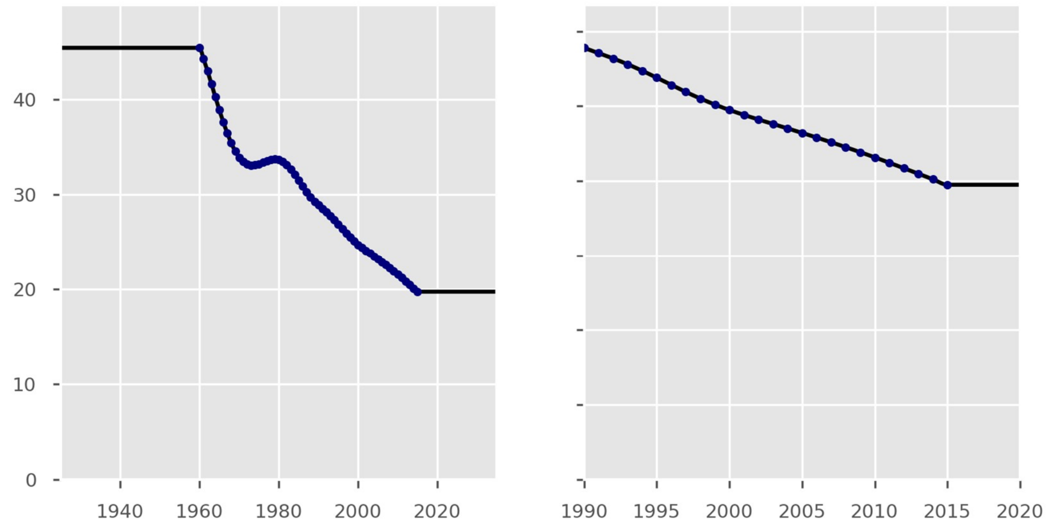

Figure S1. Crude birth rate per capita time-variant parameter. Data source: the World Bank 2016.

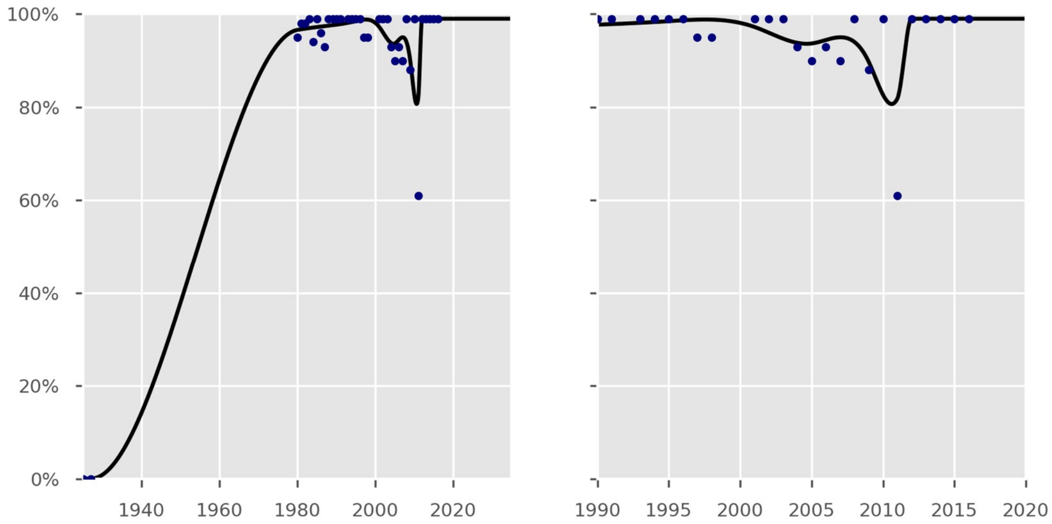

Figure S2. BCG vaccination coverage time-variant parameter. Additional data value of zero in 1927 added manually to reflect unavailability of BCG vaccination prior to this date. Data source: WHO/UNICEF.

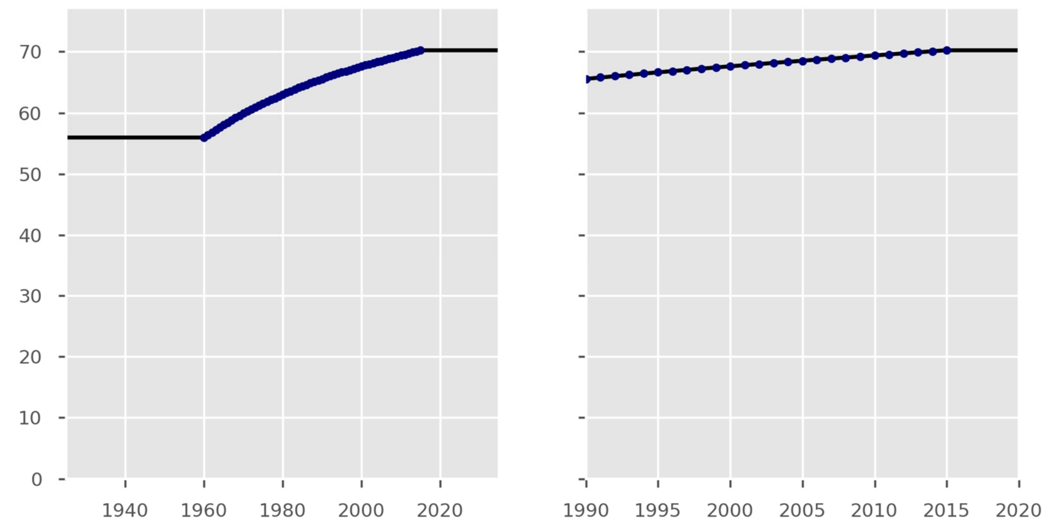

Figure S3. Life expectancy time-variant parameter. Data source: the World Bank 2016.

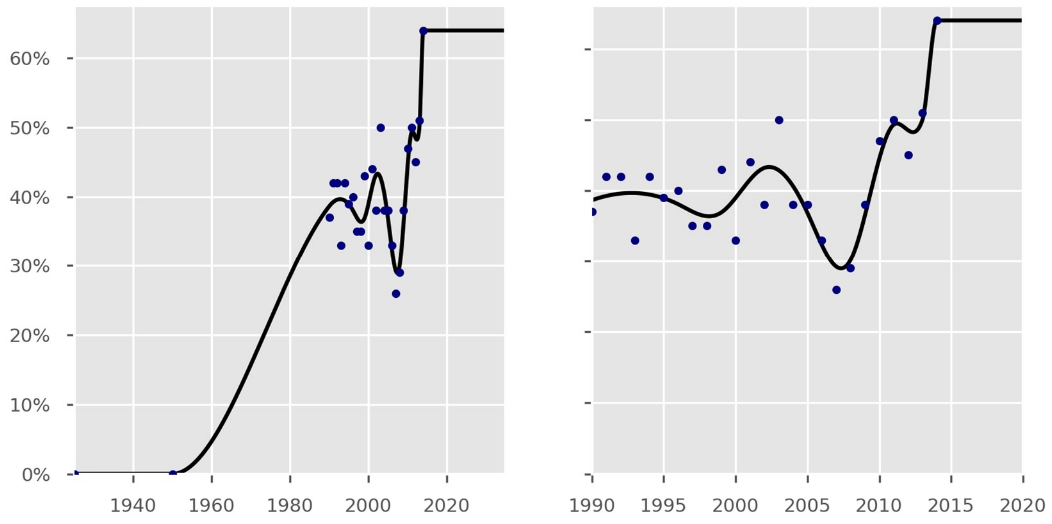

Figure S4. Case detection rate (proportion) time-variant parameter. Additional value of zero in 1950 added to reflect the unavailability of treatment prior to this date. Data source: WHO, Global Tuberculosis Report 2016. Note that data from the Global Tuberculosis Report 2016 were preferred to data from 2017, because each value is identical from 2000 to 2015 in the 2017 Report.

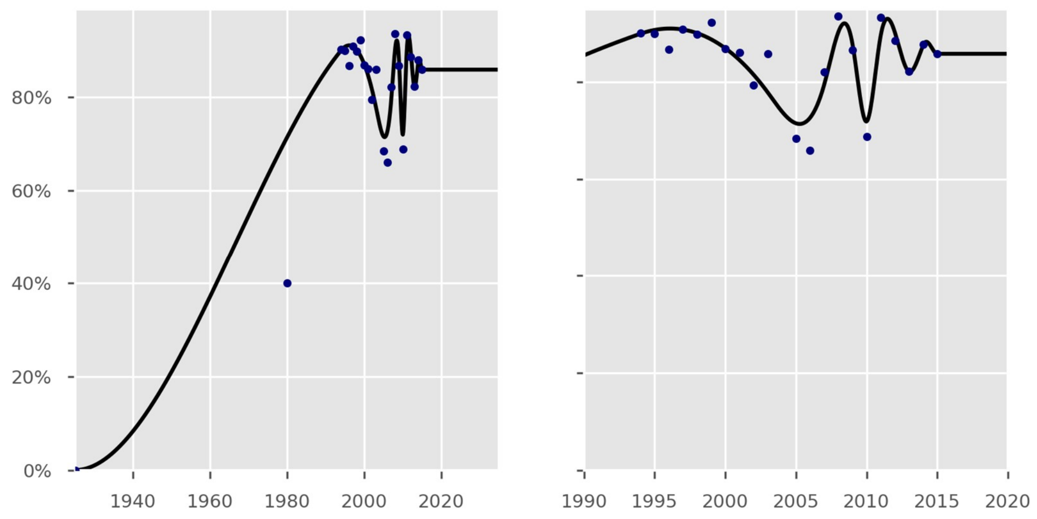

Figure S5. Time-variant parameter of the treatment success outcome for new patients. Data source: WHO, Global Tuberculosis Report 2017. Note that value of the parameter is inconsequential until the case detection rate exceeds zero in 1950 (Figure S4).

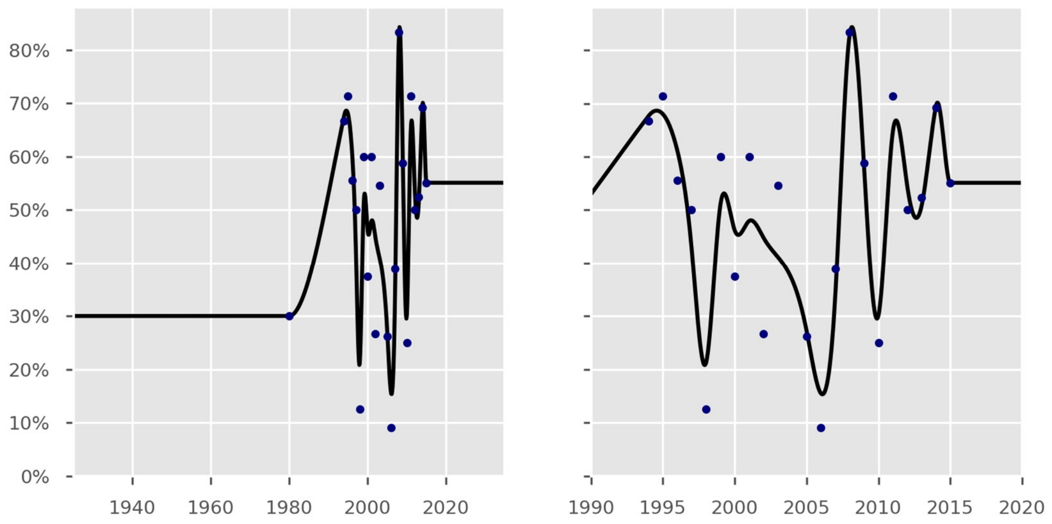

Figure S6. Time-variant parameter of the proportion of unsuccessful outcomes resulting in death for new patients. Data source: WHO, Global Tuberculosis Report 2017. Note that the value of the parameter is inconsequential until the case detection rate exceeds zero in 1950 (Figure S4). Further clarification of the meaning of this parameter is presented in Section 13.

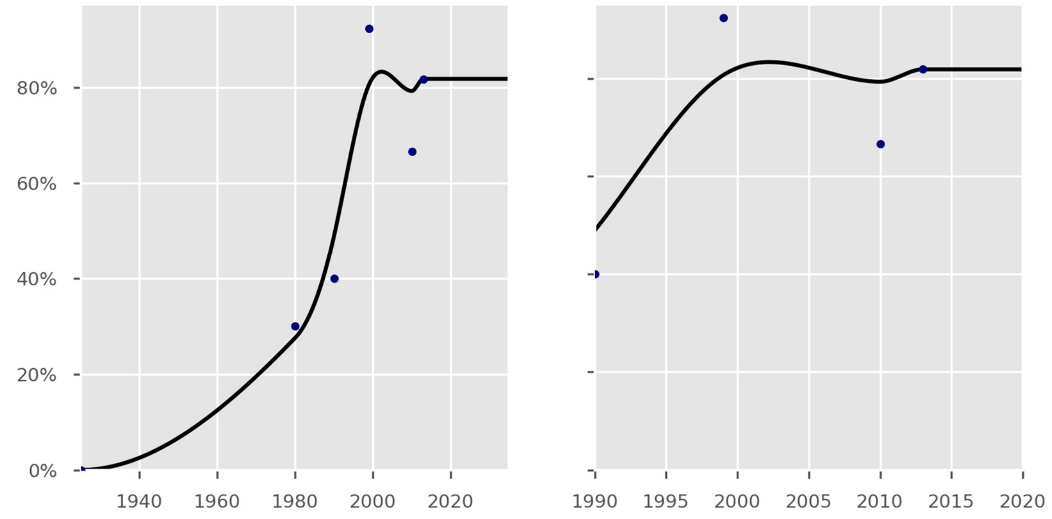

Figure S7. Time-variant parameter of treatment success outcome in previously treated patients. Data source: WHO, Global Tuberculosis Report 2017. Note that the value of the parameter is inconsequential until the case detection rate exceeds zero in 1950 (Figure S4).

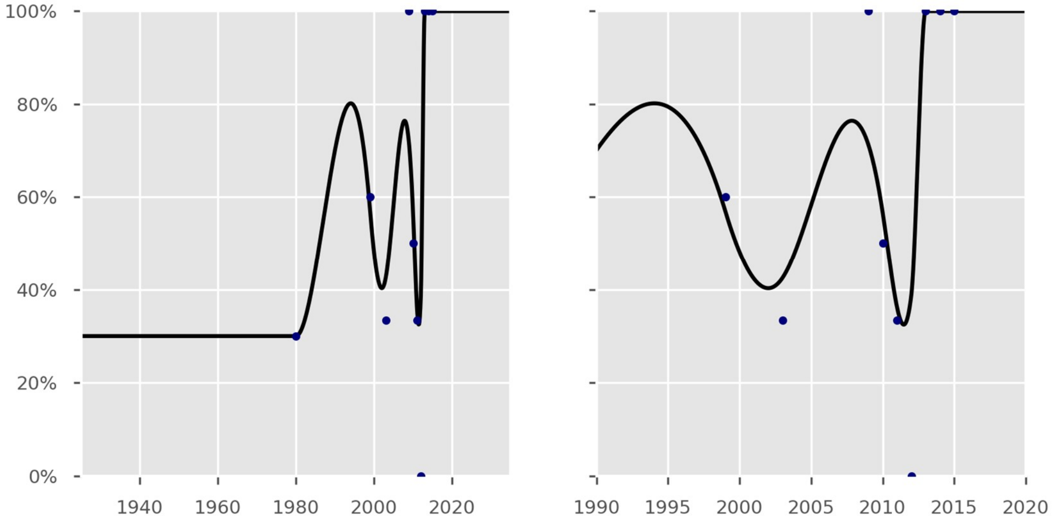

Figure S8. Time-variant parameter of the proportion of unsuccessful outcomes resulting in death on treatment for previously treated patients. Data source: WHO, Global Tuberculosis Report 2017. Note that the value of the parameter is inconsequential until the case detection rate exceeds zero in 1950 (Figure S4).

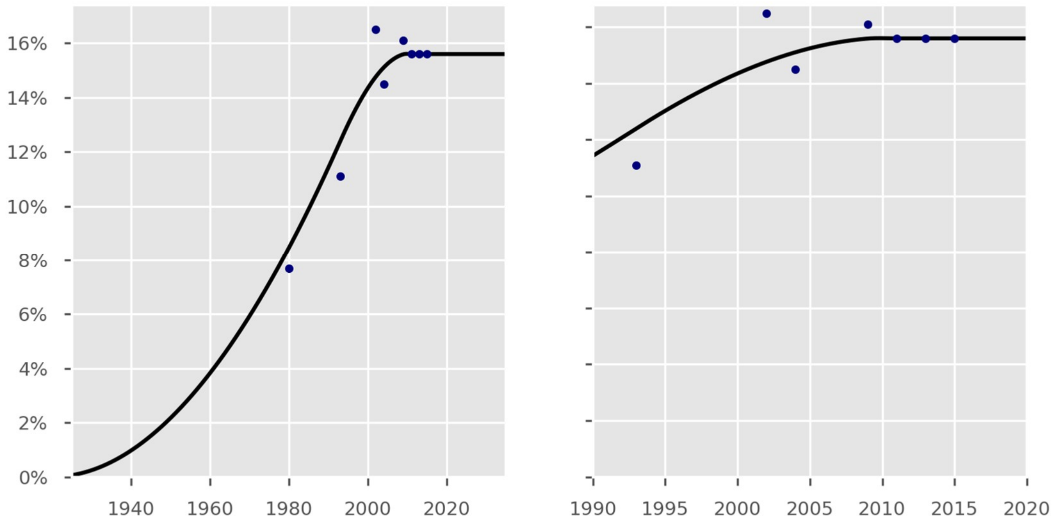

Figure S9. Simulated proportion of adult population with diabetes mellitus.

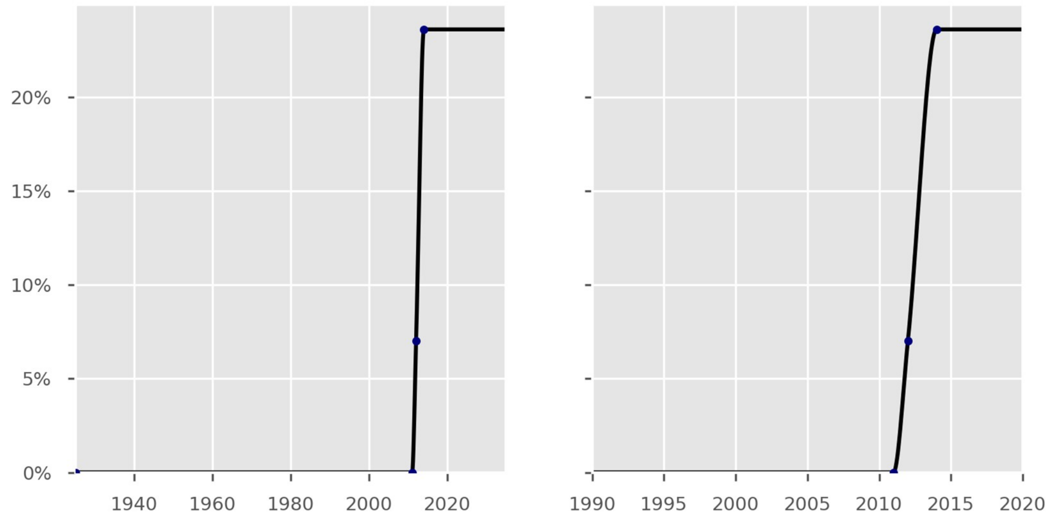

Figure S10. Time-variant parameter of the coverage of isoniazid preventive therapy in under five-year-old contacts. Data source: Fiji National Tuberculosis Program.

Constant parameters

The constant parameters (first group in Section 2) that do not vary with the calendar time through model integration are presented in Table S1 below. The rationale and review of the relevant evidence that were used to determine these parameter values are presented in the following sections.

Table S1. Constant parameter values

Parameter	*Value*	*Unit*
Latency progression parameters		
Duration in early latency (all age groups)	60	Days
Proportion of persons progressing to active TB from early latency if aged under 5 years	56	%
Proportion of persons progressing to active TB from early latency if aged 5 to 15 years	26	%
Proportion of persons progressing to active TB from early latency if aged 15 and above (including 15 to 25 and 25 and up age groups, non-diabetics only)	3	%
Rate of progression from late latency to active disease (all age groups)	0.245	% per year
Natural history of disease		
Duration of active disease if untreated	2.82 (2 to 4)[a]	Years
Case fatality of untreated smear-positive active TB	72.6 (35 to 85)[a]	%
Relative case fatality for smear-negative and extrapulmonary TB, by comparison to smear-positive TB (i.e. 20% CFR if smear-negative, 73% CFR if smear-positive)	20 ÷ 73	Multiplier
Other biological		
Effective contact rate	10.0 (5 to 20)[a]	Contacts per year
Proportion of incident cases smear-positive	33.1	%
Proportion of incident cases smear-negative	39.4	%

Proportion of incident cases extrapulmonary	28.5	%
Relative protection from further infection conferred by current latent infection	0.21	Multiplier
Relative protection from infection (i.e. multiplier applied to the force of infection) for vaccinated children whose immunity has not yet waned	0.5	Multiplier
Age after which protection against infection conferred by BCG vaccination wanes (i.e. BCG vaccination protects youngest two age groups only)	15	Years
Relative infectiousness of smear-negative cases (by comparison to smear-positive)	24	%
Relative infectiousness (i.e. contribution to the force of infection) for children (assumed as those aged 10 and below and so applied directly to the zero to 5 age group)	0.1	Multiplier
Relative infectiousness of children aged 5 to 15 (calculated as mid-point of infectiousness of those under and over 10 years of age)	0.55	Multiplier
Relative susceptibility to reinfection for previously treated patients compared to fully susceptible	0.62 (0.25 to 2)[a]	Multiplier

Comorbidity-related

Age from which diabetes is applied (i.e. the oldest of the four age groups simulated only)	25	Years
Odds ratio applied to proportion of persons progressing early to active TB if diabetic	3.11	Multiplier
Relative risk of progression to active TB from late latency if diabetic	3.11	Multiplier

Programmatic

Sensitivity of the baseline diagnostic algorithm for smear-positive cases	80	%
Relative sensitivity of baseline diagnostic algorithm	0.61	Multiplier

for smear-negative and extrapulmonary cases		
Treatment duration	6	Months
Duration infectious after commencing treatment	10	Days

Time period before patients re-start seeking care after a false-negative diagnosis of TB	3	Months
Time from presentation to treatment commencement, smear-positive and extrapulmonary cases	7	Days
Time from presentation to treatment commencement, smear-negative cases	30	Days

Other miscellaneous

Model run start time	1845 (1830 to 1920)[a]	
Modern population targeted in 2016	892,000	Persons
Infectious seed (i.e. first calibration run starts with 190,000 susceptible persons and three active patients, with total starting population varied but proportion infectious at commencement remains constant)	3 ÷ (3 + 190,000)	Proportion

[a] Epidemiological uncertainty parameter varied during calibration, presented as: calibrated value (lower limit of permitted range to upper limited of permitted range). Note that references to the literature that are used to inform parameter values are not presented here, as the rationale for the choice of values is presented in detail in the model description below.

Epidemiological uncertainty parameters

We restrict the parameters that can be varied during calibration to TB-specific parameters that would otherwise remain constant throughout model runs. This behaviour is chosen so that the uncertainty calculations capture the considerable epidemiological uncertainty in such parameters, whereas the programmatic parameters are fitted to the best available data for each time point available. Similarly, the intervention-related and economic parameters are held constant throughout the epidemiological uncertainty simulations. The uncertainty parameters are indicated by providing a lower and upper value of the uncertainty range that may be explored. Each uncertainty parameter is varied between each simulation using a Metropolis–Hastings algorithm. When running uncertainty, the base case (i.e. without interventions other than vaccination and IPT in under five years of age applied) is run to completion, and acceptance is determined based on this simulation only.

Prior distributions

Figure S11 presents the prior distributions used for the five calibrated parameters. The parameters of the gamma and beta distributions were obtained by specifying the values of the mean and standard deviation, and the shape and scale parameters were computed accordingly. We used the following distributions:

- Run start time: uniform (1830, 1920)
- Relative susceptibility after treatment: gamma (2.8, 0.36). Mean = 1 and sd = 6
- Effective contact rate: uniform (5, 40)
- Case fatality of untreated smear-positive TB: beta (5.8, 2.5). Mean = 0.7 and sd = 0.15
- Duration of untreated active disease: gamma (36, 0.083). Mean = 3 and sd = 0.5

Likelihood

The likelihood is obtained by assuming that the country TB incidence at a given time point is normally distributed around the predicted TB incidence provided by the model for this same time point. As the time period 2010–2016 is used for calibration and because the WHO provides yearly estimates for TB incidence, this means that the likelihood is made of seven components that are all multiplied together.

Posterior distributions of the parameters

Figure S12 presents the posterior distributions of the five calibrated parameters.

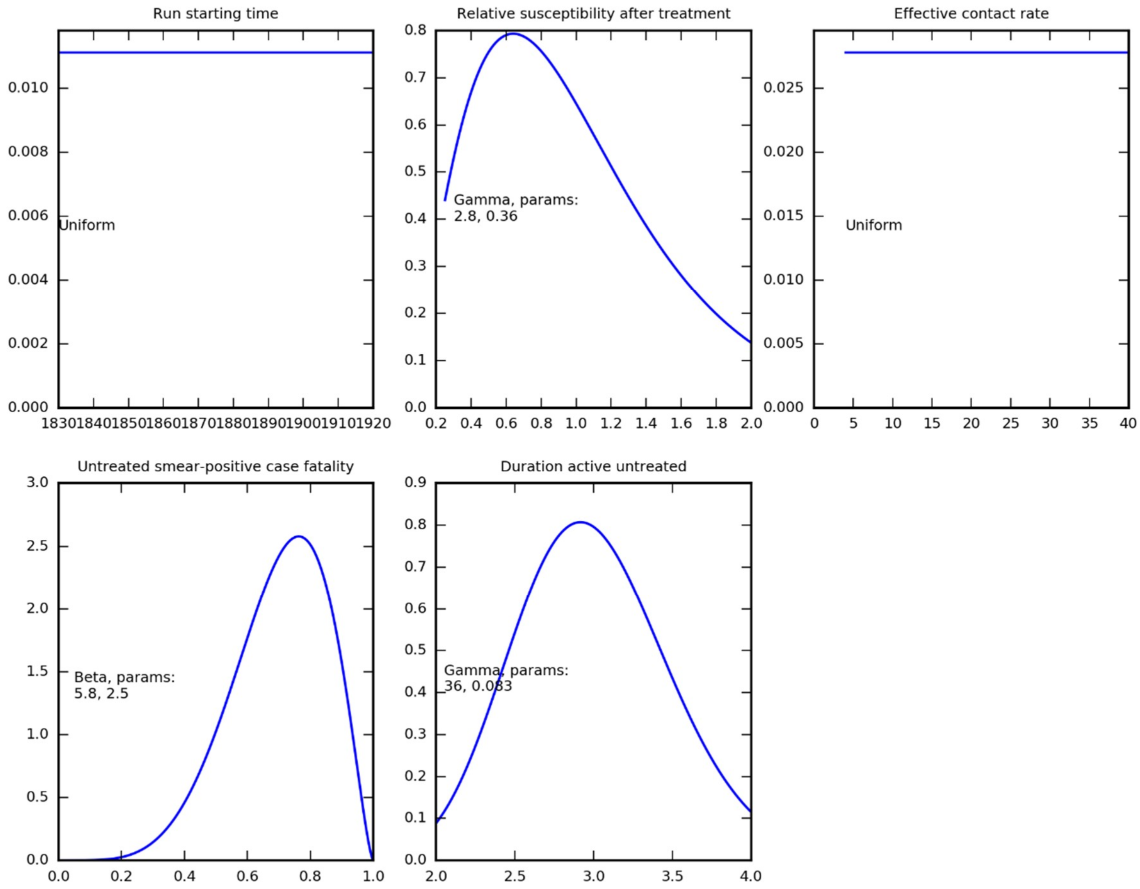

Figure S11. Prior distributions for uncertainty parameters.

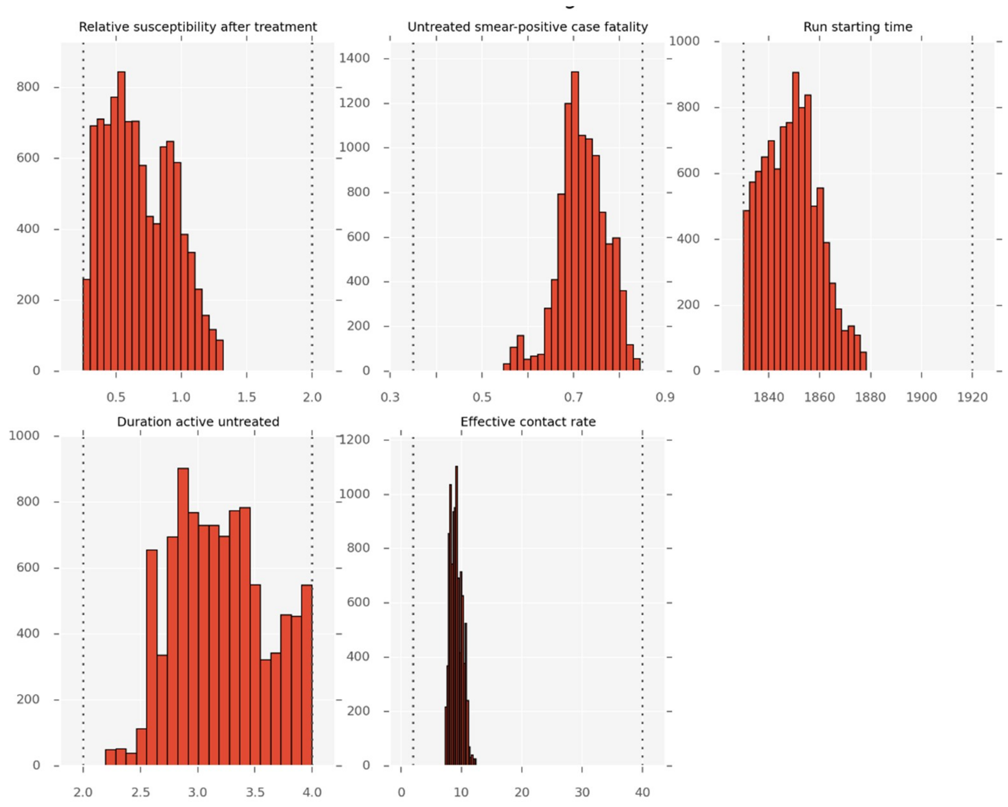

Figure S12. Posterior distributions for uncertainty parameters.

Model initialisation

At model commencement, persons are assigned to the fully susceptible and active compartments only. The proportion of persons assigned to active TB remains constant throughout all the calibration model runs, while the total starting population is algorithmically varied to target a specified modern population. This is achieved by first calculating the ratio of the simulated modern population and the targeted modern population at each integration run. Next, the starting population is multiplied by the reciprocal of this ratio. For example, if a run over-shoots the modern population by 1%, the starting population is multiplied by 100/101 (revising the starting population down by approximately 1%).

Demographic parameters

For simulating births into the model, we assign a time-variant parameter using publicly available crude birth rate data from the World Bank [3]. The birth rate is interpreted as crude births per 1000 population (as is the case for the World Bank data) and so is multiplied by the total population of the model divided by 1000 to determine the overall rate of births entering the susceptible compartment (Figure S1). This is then distributed between vaccinated and unvaccinated births according to Bacillus Calmette–Guérin (BCG) coverage at the time point simulated, to reflect the coverage of neonatal vaccination.

Simulating non-TB-related deaths or the background death rate is achieved with an analogous method. Life expectancy is obtained from publicly available data from the World Bank [3], and a time-variant parameter is fitted to these data points (Figure S3). Then, population deaths are calculated as the reciprocal of this value and applied equally to every model compartment, including all the compartments for patients with active TB (in addition to TB-specific mortality), all risk groups, and all age groups.

Susceptibility/immunity to infection

The degree of susceptibility or immunity to infection among different population groups given their vaccination status, *Mtb* infection-related compartment, age, and treatment history is one of the most uncertain yet important parameters in TB epidemiology. We consider homogeneous susceptibility/immunity to *Mtb* across risk groups and age groups, assuming that the differing rates of TB disease in these groups are related to the other factors described below. By contrast, BCG vaccination and latent infection with *Mtb* are considered to influence the likelihood of infection after exposure to an infectious individual. The effect of BCG vaccination on immunity is described under the section on BCG vaccination below. The effect of prior latent infection on reinfection is estimated from a review of cohort studies comparing those with evidence of prior *Mtb* infection to those without such evidence among persons exposed to infectious TB. This study found that the rate of new episodes of TB disease in those with prior latent infection was 0.21-fold that in those without. Since the reactivation of past infection would lead to even lower values, we use this estimate as our parameter value for the relative rate of reinfection among currently latently infected persons (by comparison to the rate of infection in fully susceptible persons) [4]. Note that this only applies to the late latent compartment, as the reinfection of those in the early latent compartment will not affect their risk of progression to active disease, as these persons are already conferred the highest risk of progression to TB.

Subsequent episodes of TB disease after recovery from a prior episode are known to occur at a higher rate than that in the background population [5]. However, whether this is due to an increased host susceptibility to reinfection incurred by the previous episode or to underlying heterogeneity in rates of reinfection is unclear. In the latter case, it is feasible that recovered or treated individuals have equivalent or even decreased susceptibility to future infection than those who have never been infected [6]. We use a multiplying parameter to allow for individuals who have previously been treated for active TB to be assigned a different level of susceptibility to subsequent infection from those who have not. This multiplier is applied in addition to the parameters described above for latent infection and vaccination, such that the total level of susceptibility is the product of these two parameters. This parameter is varied during uncertainty, with its prior distribution presented in the second panel of Figure S11. For example, previously treated and latently infected persons would both have a relative risk of 0.21 compared to fully susceptible persons where this parameter is set to one.

Model of *M. tuberculosis* infection and progression

General approach and compartmental structure

Our approach to quantifying rates of progression from latency to active disease is described in detail in two published journal articles. The first uses TB surveillance data from a very low transmission setting to estimate rates of active disease following a defined index exposure to an infectious case and evidence of exposure [7]. The second article uses the reactivation profiles from this study and from a similar epidemiological investigation [8] to determine the

ideal compartmental structure and progression parameters to accurately capture progression to active TB following infection [9]. This second publication found that two latency compartments placed in series are highly effective at reproducing epidemiological observations when appropriately parameterised, and appropriate parameter values are provided. Specifically, following infection, all the latently infected individuals first progress to a high-risk "early latent" compartment, from which they may progress rapidly (within months) to active TB. Alternatively, these individuals may "stabilise", transitioning to a low-risk "late latent" compartment, from which the progression to active TB occurs much more slowly. This configuration is also consistent with that used or recommended in other important TB modelling papers [10]. This approach requires the estimation of three epidemiological parameters: the time period spent in the early latent compartment, the proportion of individuals progressing to active disease from the early latent compartment, and the rate of progression from the late latent compartment to active disease (values presented in Table S1). Then, these are converted into three rates for early progression, stabilisation, and late progression.

Organ manifestation

We distinguish three types of organ manifestation of active TB: smear-positive pulmonary, smear-negative pulmonary cases, and extrapulmonary cases. We assume that the proportion of notified TB cases that are pulmonary or extrapulmonary, along with the proportion of pulmonary cases that are smear-positive, are constant over time. We calculate this value as the total number of notifications reported by each organ manifestation, and use this constant value for the proportion of incident cases by organ manifestation.

Natural history

Once TB disease has manifested, natural history parameters are first set to reflect outcomes in the absence of detection by the health care system. The total rates of progression to either death or spontaneous recovery are set to equal the reciprocal of the time period of untreated active disease. Next, a proportion of this total rate is then allocated to each of the two possible outcomes (death and spontaneous recovery) according to the case fatality rate (a proportion) for smear-positive TB. The smear-negative and extrapulmonary case fatality rates are then both set to be proportionately lower than the smear-positive case fatality rate by a relative multiplier parameter that remains constant throughout model runs. That is, the rate of untreated death is equal to the reciprocal of the time period untreated multiplied by the case fatality rate, while the rate of spontaneous recovery is equal to the reciprocal of the time period untreated multiplied by the complement of the case fatality rate. Parameter values and uncertainty ranges are estimated from a detailed review of pre-chemotherapy era outcomes [11].

Case detection

Obtaining the case detection ratio

The case detection rate is a time-variant parameter and is a fundamental parameter determining the performance of TB programs at the country level. Estimates of the case detection rate are provided by the World Health Organization in the Global TB Report [12]. It is important to note that this "rate" is actually a ratio or proportion, and is referred to as the case detection ratio hereafter. Specifically, the case detection ratio is the proportion of all the active cases that are detected during their episode of disease, which is also expressed as the ratio of notifications to incidence in the geographical region considered. Variations in its magnitude have major impacts on disease burden that may only be realised decades later. Effective medical treatments for active TB only became available from the 1950s onwards. Since there was no effective treatment that improved on the natural history of the disease, we apply an effective case detection ratio prior to that time of zero. As with other time-variant parameters, case detection ratio data are loaded from the Global TB Report data.

Adjustment to the case detection ratio for decentralisation

After the case detection ratio time-variant parameter has been defined as described above, it is next adjusted for the decentralisation intervention. This is described in detail in Section 17, and has the general effect of narrowing the gap between the actual, loaded case detection ratio and an idealised perfect case detection ratio.

Adjustment to the case detection ratio by organ status

We assign different values to the case detection ratio according to organ status, because it is likely that smear-positive, smear-negative, and extrapulmonary cases are diagnosed by the health care system at different rates. Smear-positive cases may be targeted by the health system, and may be easier to diagnose with the tools locally available (smear microscopy in particular). Moreover, several interventions may act to close this diagnostic gap between smear-positive and smear-negative or extrapulmonary cases. For example, GeneXpert improves the possibility of detection with smear-negative TB cases relative to smear microscopy and clinical judgement, partially closing this detection gap, as described below.

In our model, the rate of detection for smear-positive cases is increased, while the rates of detection for smear-negative and extrapulmonary cases are decreased. This is calculated such that the smear-negative and extrapulmonary cases have a case detection ratio that is lower than that of the smear-positive cases by a constant specified ratio, while the weighted average case detection ratio for all the forms of TB remains equal to the unprocessed case detection ratio originally estimated.

Missed diagnosis and the accuracy of the diagnostic algorithm

In order to explicitly simulate patients who presented for care but whose diagnosis was missed, a flow rate that transitions patients from the active untreated compartment to a missed diagnosis compartments is simulated. Patients whose diagnosis has been missed suffer outcomes applicable to those in the active treatment compartment in terms of death and spontaneous recovery, but do not re-start seeking treatment for a limited period of time. To

achieve this, it is considered that case detection is linked to the diagnostic algorithm, as missed cases are proportional to its complement. That is:

$$\begin{aligned} &rate\ of\ case\ detection \times algorithm\ accuracy \\ &\qquad = rate\ of\ missed\ diagnosis \times (1 - algorithm\ accuracy) \end{aligned}$$

The adjustment process for organ status described for the rate of case detection is applied in the same way to the sensitivity of the baseline diagnostic algorithm parameter in order to simulate a greater proportion of smear-positive persons being detected rather than missed under baseline conditions through clinical assessment and smear microscopy (i.e. prior to implementation of novel diagnostics, such as GeneXpert).

Treatment outcomes

Determining aggregate outcomes

Treatment outcomes are derived from the Global TB Report, which reports outcomes for patients differently by strain, HIV status, sputum smear status, and treatment history, as well as changing its reporting approach for some categories after 2011. All the reported treatment outcomes are grouped into three broad categories for model interpretation: 1) treatment success, 2) death during treatment, and 3) survival with unfavourable outcomes (i.e. any outcome other than success or death, with the terminology for these pathways having changed over time).

For patients up to and including 2011, patients achieving treatment completion or cure (the latter for smear-positive patients only) are classified as treatment success, patients dying on treatment are classified as such, and patients reported as default or failure are classified into the third category. Up to 2011, relapse cases are included with retreatment cases in Global TB Report data. After 2011 for non-MDR-TB patients, loss to follow-up and failure are classified as unfavourable outcomes (as opposed to default and failure up to 2011). After 2011, relapse cases are included with new cases in official data, in contrast to the situation up to 2011. As there is no reliable way to account for this change, it should be noted that outcome parameters are fit to data that changes its characteristics after 2011, although this change is very small in magnitude.

Calculation of proportions

Once the total outcomes have been classified into the three categories described above, the proportion of patients falling into each category is calculated. Then, two time-variant proportion functions are defined. The first is the treatment success proportion, for which the numerator is successful treatment outcomes (cure and completion), and the denominator is all treatment outcomes (figures S5 and S7). The second is the proportion of unsuccessful outcomes resulting in death, for which the numerator is death outcomes, and the denominator is all the treatment outcomes other than success.

Then, time-variant functions representing proportions (limited to range zero to one) are calculated for these quantities. Then, the proportion of defaults is calculated as the proportion of non-success outcomes multiplied by the proportion of unsuccessful outcomes resulting in death. Using this approach, all the treatment outcomes must consistently remain non-negative, and the three possible outcomes always sum to one.

Treatment history

All the compartments are duplicated according to whether patients have been previously treated or not. The only exception is for the fully susceptible unvaccinated compartment(s), which is not duplicated, as there is no equivalent compartment representing fully susceptible individuals in the previously treated stratum. This is because previously treated individuals are assigned a modified level of immunity/susceptibility to future episodes of infection.

Age differences

Risk of progression to active disease

The natural history of childhood TB was described in detail by Marais et al. in their 2004 publication that considered historical publications on this topic in detail [13]. The period from 1920 to 1950 was particularly informative for the study of the natural history of TB, as chest radiography had become available, but effective treatment to modify the natural history had not. Although quantitative estimates of the risk of progression to active disease and subtypes are presented, we prefer the publications by Sloot et al. and Trauer et al., as these two modern estimates employ formal survival analysis techniques and are highly consistent with one another [7,8]. Moreover, after modification of the progression rates for BCG-vaccination, these estimates are likely to be consistent with those of Marais et al.

Infectiousness

Although children are often considered non-infectious, some groups of children are likely to have the potential to transmit infection, including adolescents who more often have adult-type reactivation TB. Other potentially infectious paediatric groups include those with pulmonary cavitation, positive smears for acid fast bacilli (AFBs), laryngeal involvement, widespread pulmonary disease, and suspected congenital TB (which tends to be more extensive). However, younger children, including those with primary pulmonary TB, are unlikely to be infectious because of their low bacillary load [14] and inability to generate a sufficiently forceful cough [15]. This is supported by the observation that an infectious adult was consistently identifiable in outbreaks of TB in orphanages and children's hospitals, while such outbreaks did not occur when only a child was identified with active TB. Eight case reports of transmission from children were identified in a 2001 review, which included four children aged under one (including two with congenital TB), with the others aged three, five, seven, and nine. However, the extent of transmission was either limited or incompletely described in most of the six reports considering children aged five and below. Unpublished data recording low rates of tuberculin skin test (TST) conversion in the staff of paediatric hospitals also support this contention [16]. For these reasons, we reduce the infectiousness of children by one order of magnitude (i.e. 0.1 times that of adults) and apply this to estimates of the under-five age group. Although we maintain the division into smear-negative/smear positive and extra-pulmonary in this age group to reflect the same qualitative status as in the adult age group, we acknowledge that all of the members of this group are likely to be smear-negative most of the time, and use the (0.1-fold) multiplier to reflect this. For the age group aged 5 to 15, we assume that approximately half of these patients will have adult-type and half child-type manifestations of disease, resulting in a weighted average of 0.55 times the infectiousness of adults for this group.

Case fatality

The case fatality rate (a proportion) for children with miliary TB was estimated at 14% in one case series [17], which is considerably greater than the estimate of 3.5% for all cases from one meta-analysis [18]. However, this is clearly not a direct comparison, as this higher rate was observed only in a subgroup of paediatric cases, and may well be offset by lower case

fatality rates in the other subgroups, such as tuberculous lymphadenitis. Moreover, these estimates primarily consider patients under treatment, and so cannot be used to estimate the case fatality of untreated TB (which is of greater relevance to our model parameters).

Conclusions and model implementation

We use the age-specific progression proportions reported by Trauer et al. and are currently undertaking further work to refine these parameters. Although difficult to quantify, the infectiousness of persons aged under 10 is lower than those of older ages, and we apply a 10-fold reduction as described above. Case fatality rates are not currently modified for age, although we continue to seek evidence to quantify this.

Diabetes

Implementation

We estimate the relative risk or odds ratio for developing TB in patients with diabetes by comparing the risk of disease in affected patients to the risk for patients without diabetes. Ideally, a relative risk or hazard would be applied to the rate of late progression from late latency to active disease, while an odds ratio would be applied to the proportion of patients in early latency who progress to active disease rather than stabilising to late latency. For individuals in early latency, we do not adjust the duration of early latency for persons with comorbidities. Instead, we only adjust the duration of early latency for the proportion of patients undergoing early progression to active TB. We apply an estimate of the odds ratio for progression to active disease associated with the comorbidity to the original estimate for the proportion progressing, which is age-specific in the age-stratified models. We term the proportion of persons in early latency progressing to active TB in the absence of comorbidities "p_n", such that the odds of progressing is equal to $p_n / (1 - p_n)$. Similarly, the proportion progressing in the presence of the comorbidity is termed "p_c", with associated odds $p_c / (1 - p_c)$. Therefore, if the odds ratio associated with the comorbidity is termed "OR":

$$\frac{p_c}{1 - p_c} = OR \times \frac{p_n}{1 - p_n}$$

Solving for p_c gives:

$$p_c = \frac{p_n \times OR}{p_n \times (OR - 1) + 1}$$

This formula is used to calculate the rate of progression in the presence of diabetes. It has the advantages of producing a similar modification in p_c to that achieved with a relative risk calculation if the odds ratio (OR) is relatively small, but having a ceiling value at one for large ORs. As this is a proportion that is used to determine the rates of flows from early latency, the rate of progression from early latency to active disease and to late latency are:

$$early\ latency\ to\ active\ disease\ progression\ rate = \frac{p_c}{duration\ early\ latency}$$

$$early\ to\ late\ latency\ progression\ rate = \frac{1 - p_c}{duration\ early\ latency}$$

Quantification of effect

Several epidemiological studies have found a greater rate of incident TB in patients with diabetes across a range of contexts [19,20]. Observations from such individual studies include a generally similar or greater rate of bacteriologically-confirmed disease than for all forms [21] and similar increases in risk associated with recent and reactivation disease [22]. One study reported a slightly lower strength of association, but recruited controls with medical conditions that are potentially weakly associated with TB [23]. A limitation of several of these studies is that TB itself is known to be associated with transient impairment to glucose metabolism [24,25], although the above studies varied as to whether they considered previously diagnosed diabetes or performed glucose metabolism testing at the time of

diagnosis. Several of the above-mentioned studies were included in a meta-analysis of the association of diabetes on TB published by Jeon et al. in 2008 [26]. This study found a relative risk for diabetes of 3.11 (95% CI 2.27 to 4.26) from a random effects meta-analysis of three cohort studies. To apply the modification in the rates of progression to active disease due to diabetes, we adopt the approach outlined above, estimating both the odds ratio for early progression and the relative risk for late progression at 3.11.

Impact of diabetes on TB epidemic

The following figure presents the scale-up functions that were used to simulate different levels of diabetes prevalence in the future population of Fiji.

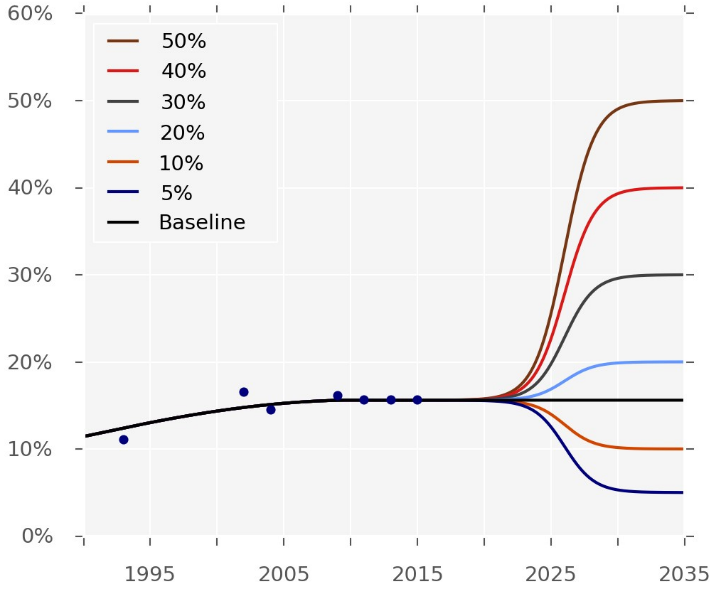

Figure S13. Scale-up curves used to vary future diabetes prevalence in Fiji.

Additional outputs to illustrate the underlying dynamics of the model

Age distribution of the Fijian population

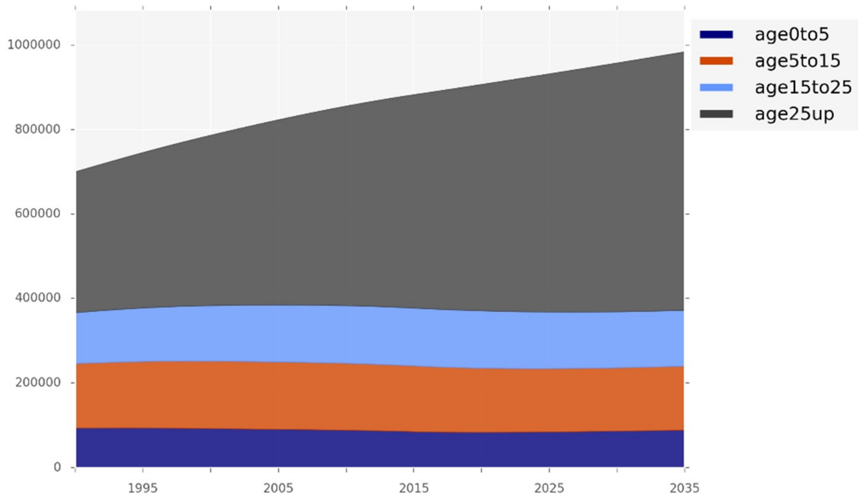

Figure S14. Age distribution of the simulated Fijian population over time.

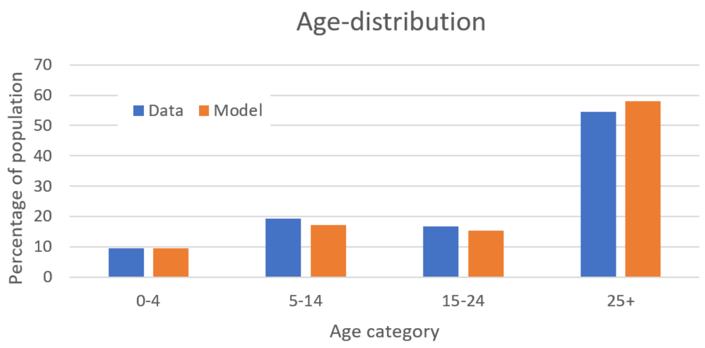

Figure S15. Comparison between the simulated age distribution and the data reported by the Population Division of The United Nations for year 2017.

Disease burden estimates by age groups

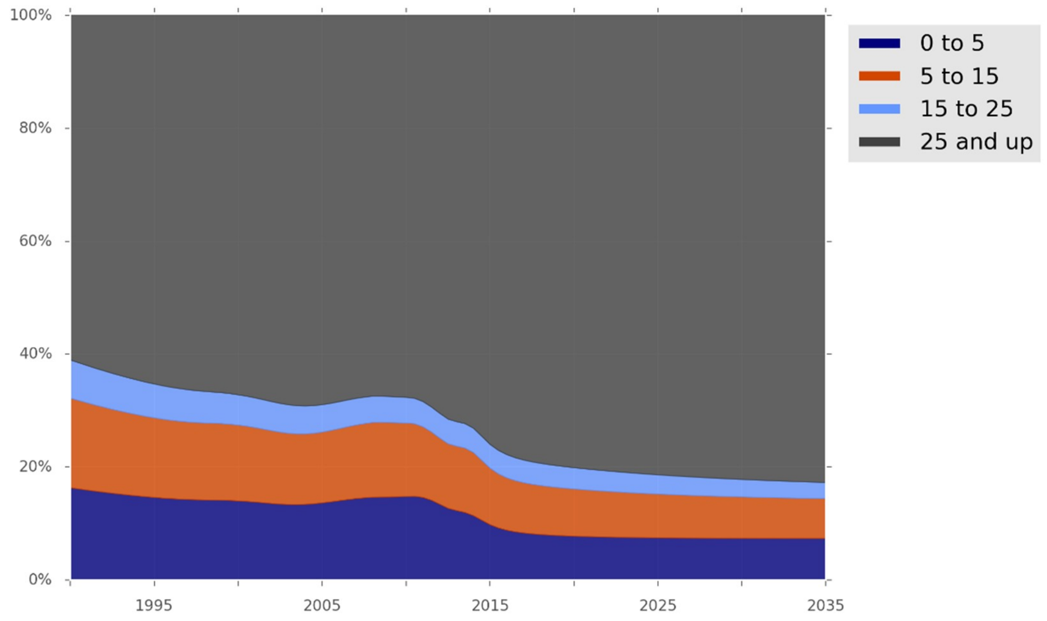

Figure S16. Estimated distribution of TB incidence by age group over time.

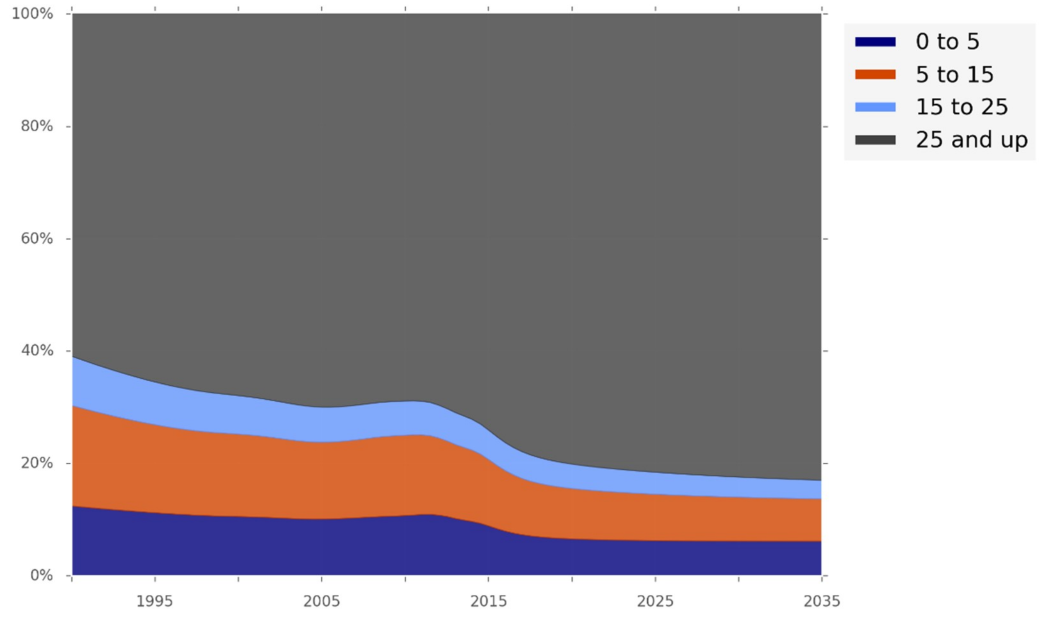

Figure S17. Estimated distribution of TB notifications by age group over time.

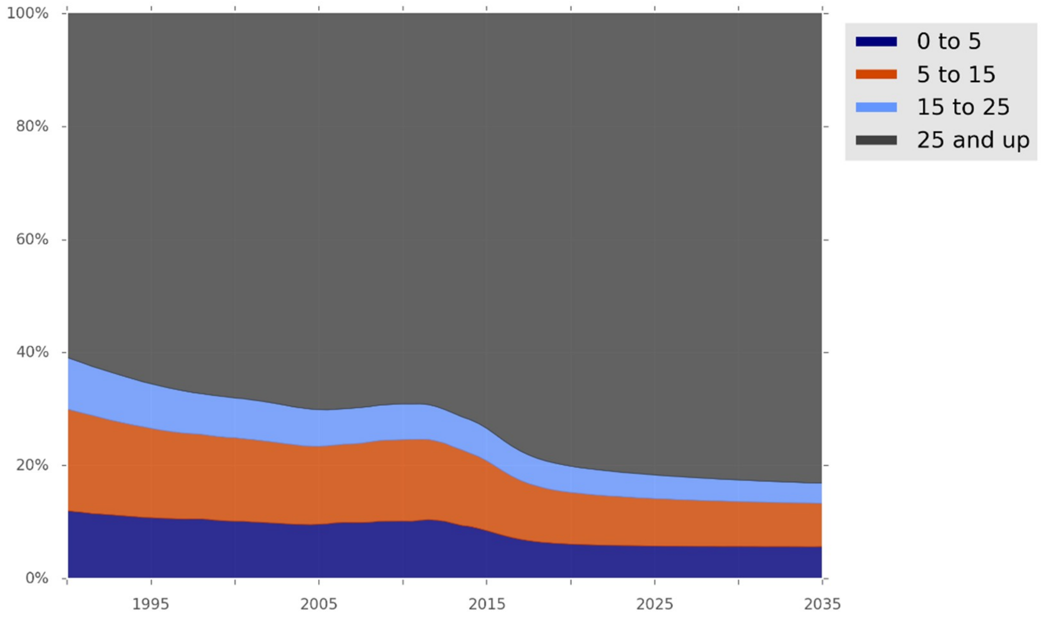

Figure S18. Estimated distribution of the number of TB deaths by age group over time.

Profile of incident TB: fast progression versus late reactivation

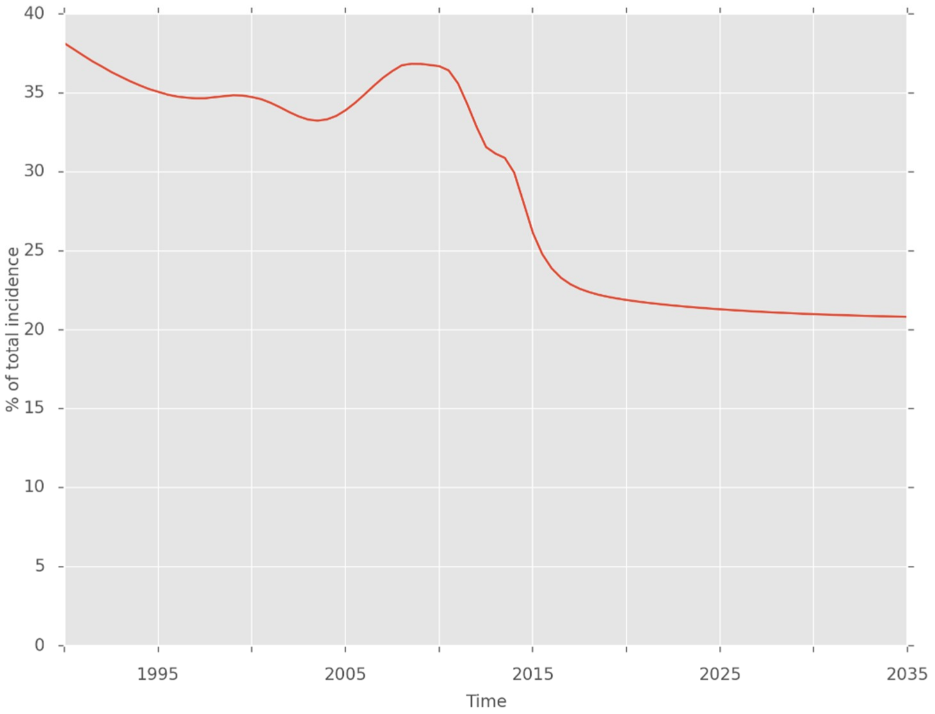

Figure S19. Percentage of incident TB that is due to fast progression rather than late reactivation.

Support for patients under treatment

Implementation

Although evidence is variable and past research has been inconsistent, it is likely that carefully constructed, context-specific, multifactorial interventions to support patients in treatment are effective in improving patient adherence and treatment outcomes. For model implementation, unfavourable treatment outcomes are reduced to 0.43 (95% CI 0.21 to 0.89), multiplied by the coverage of the intervention times the pre-intervention values, which are consistent with findings from a study in Senegal [27]. This is intended to reflect a comprehensive change to how patients are supported during their treatment across the country.

Results

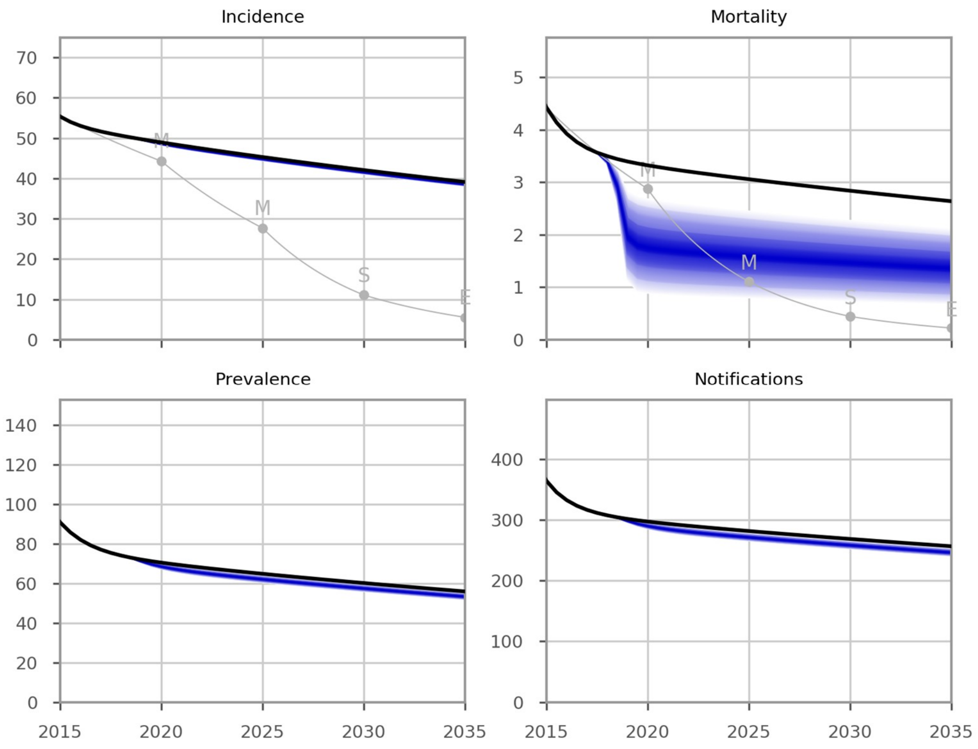

Figure S20. Effects of treatment support with intervention uncertainty.

Decentralisation

Implementation

In the context of Fiji, the decentralisation of clinical care for patients with active TB represents a comprehensive intervention to bring high-quality TB care to patients in their local communities. The effect of this intervention, when fully implemented, is to entirely eliminate all the geographical barriers to accessing curative care for any patient with TB in any geographical location in the country. Conceptually, this means that patient-related delays to starting treatment are considerably reduced, such that case detection is faster and more consistent across the country.

Given the logistical difficulties, few or no studies have considered an intervention consisting of the expansion of health facilities in the context of medical research or a randomised controlled trial. Ecological studies give some sense that the decentralisation of TB treatment facilities is likely to improve case detection rates. For example, observations from Armenia indicate that TB clinics located near urban centres and examining more presumptive cases of TB are likely to have greater TB notification rates [28]. Similarly, geospatial modelling from a remote zone of Ethiopia indicates that notification rates are higher in areas with closer proximity to TB health care [29]. Studies from various contexts have found that treatment from decentralised, community-based, or patient-centred care centres, potentially involving outreach visits by health extension workers, is as effective as clinic-based management [30,31]. Together, this evidence implies that support for community-based TB treatment, along with an increase in the number of health facilities available, could improve case detection rates.

As passive case finding remains the basis for TB detection in most countries worldwide, we consider an "idealised" case detection rate to be equal to that estimated by the best performing TB programs reported in the WHO's Global TB Report. Low-burden, high-resource countries, along with the best-performing high-burden countries, are able to consistently achieve case detection rates of 80% to 90% for periods of several years, suggesting that this may be an upper limit for case detection through passive strategies alone. Therefore, this intervention is implemented in the model by increasing the case detection ratio from its baseline value towards an idealised case detection ratio, which by default is set to 85%. To estimate the adjusted case detection rate, the gap between the current case detection ratio and the ideal is decreased by the coverage of the intervention. That is:

$$\begin{aligned} &\mathit{estimated\ case\ detection\ ratio} \\ &\qquad = \mathit{previous\ case\ detection\ ratio} \\ &\qquad + (\mathit{ideal\ case\ detection\ ratio} - \mathit{previous\ case\ detection\ ratio}) \\ &\qquad \times \mathit{coverage\ of\ intervention} \end{aligned}$$

If the current case detection ratio is greater than the ideal case detection ratio at any time point during integration, the intervention is applicable at that time and is not applied. Note that this intervention is applied before adjustments are made for active case finding, which

may further modify the effective case detection rate modelled if both are implemented together.

Results

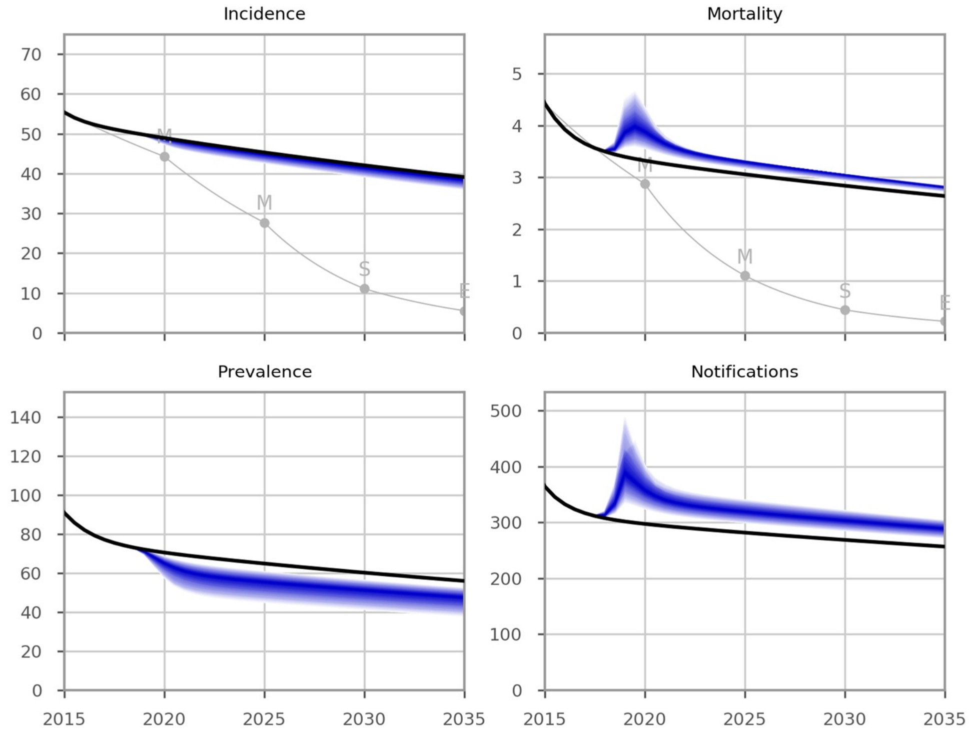

Figure S21. Effects of decentralisation on TB epidemic with intervention uncertainty.

GeneXpert replaces smear

Implementation

Background

Sputum smear microscopy has been the mainstay of TB diagnostics since the 1850s, although the test is known to have sub-optimal sensitivity, particularly for paucibacillary cases (such as HIV-associated TB, paediatric TB, and extrapulmonary TB). Meanwhile, mycobacterial culture techniques have the disadvantage of a substantial delay in results that could influence the availability of clinical decisions [32] and the technical complexity of maintaining a functioning laboratory service.

GeneXpert (Cepheid Inc, Sunnyvale, California, USA) is the current leading technology in the molecular diagnosis of TB. It was endorsed by the WHO with unprecedented speed in December 2010 as the initial diagnostic for HIV-associated TB and where high rates of drug-resistant TB are present [33]. Despite uncertainties as to the ideal approach to implementation, South Africa has led the way in deploying GeneXpert devices from 2011 to 2013, deploying 314 such devices by 2017 and being responsible for over half the global cartridge utilization [34].

Time to treatment initiation

Although GeneXpert has the potential to provide results within two hours of specimen submission, it remains important to assess the real effect on accelerating patients' progression through the treatment cascade. Reasons for a longer actual time to diagnosis or change in clinical decisions may include a preference for delaying specimen processing until a sufficient number of samples are received at a clinic.

A 2013 Cochrane review found a significantly shorter time to detection for GeneXpert compared to culture methods [35]. Moreover, there is evidence of decreased time to treatment initiation for patients with drug-susceptible TB compared to other approaches. The large multi-centre study considering the implementation of GeneXpert across six countries in three continents found a median time to detection of *Mtb* of zero days for GeneXpert, one day for smear microscopy, 16 days for liquid culture, and 30 days for solid culture [36]. However, time to treatment commencement for smear-negative, culture-positive patients decreased from a median 56 days to five days, while for smear-positive patients, a non-significant reduction from four to two days occurred. A study in Johannesburg (641 presentations with possible TB, 69% HIV-infected) found delays to treatment initiation of zero days for GeneXpert, 14 days for clinical or radiological diagnoses, and 144 days for culture-based diagnoses [37]. Similarly, a study from Saudi Arabia found results for both GeneXpert and smear microscopy were available on the same day, while cultures took a median of 22 days for results to be available [38].

The roll-out of GeneXpert technology has also been demonstrated to reduce the time taken for patients to begin treatment for MDR-TB. For example, the time to commence treatment

decreased from a median of 40 days to seven days in Latvia [39]. Similarly, in Cape Town, GeneXpert also decreased time to treatment commencement for MDR-TB by around a month, with the median time to commencement falling from 42 to 17 days [40].

A 2017 systematic review of the programmatic effect of GeneXpert included 18 studies finding a significant reduction in time to treatment, while only four did not [34].

Diagnostic accuracy for the presence of TB

In the large multi-centre study introduced above, pooled results across all the centres gave a 98.3% sensitivity for smear-positive TB, 76.9% for smear-negative (ranging from 56.3% to 100.0% by a study site) and culture-positive TB, and a specificity of 99.0% [36]. This was associated with a reduction from 39.3% to 14.7% in the proportion of patients with smear-negative, culture-positive disease who remained untreated. These estimates are close to those derived from a 2014 Cochrane review of 27 studies (including the study described above), which reported a pooled sensitivity of 98% for smear-positive TB, 67% for smear-negative, culture-positive TB, and a specificity of 99% [35,41].

However, a critical consideration is the extent of empiric treatment undertaken prior to the implementation of GeneXpert or the sensitivity of clinical assessments for TB. That is, in a setting in which the threshold for initiating empiric treatment for TB is low, implementation of a highly sensitive diagnostic may do little to increase the number of patients commenced on treatment, and so have little programmatic effect [42]. Although this is an important concern, estimating the underlying sensitivity of clinical diagnosis across a country is challenging. Several factors may influence the threshold for clinical diagnosis, with one being the underlying prevalence of TB in the population, such that rates of empiric diagnosis may be higher in high-burden countries. A 2013 meta-analysis pooled five studies that estimated the sensitivity and specificity of the clinical WHO algorithm for the diagnosis of smear-negative TB found a pooled sensitivity of 0.61 (0.55 to 0.67), but with substantial heterogeneity [43]. Clinicians in real-world settings may conceivably do better [44] or worse than such standardised diagnostic algorithms. By default, this parameter is set to 0.61.

The sensitivity of GeneXpert-based platforms may improve in the near future through the release of the GeneXpert MTB/RIF Ultra. This test is currently under evaluation [45], but is anticipated to significantly improve the sensitivity of GeneXpert for paucibacillary forms of TB (including children, HIV-positive, and smear-negative culture-positive cases) with the manufacturer claiming levels of sensitivity that approach those for liquid culture [46].

Results

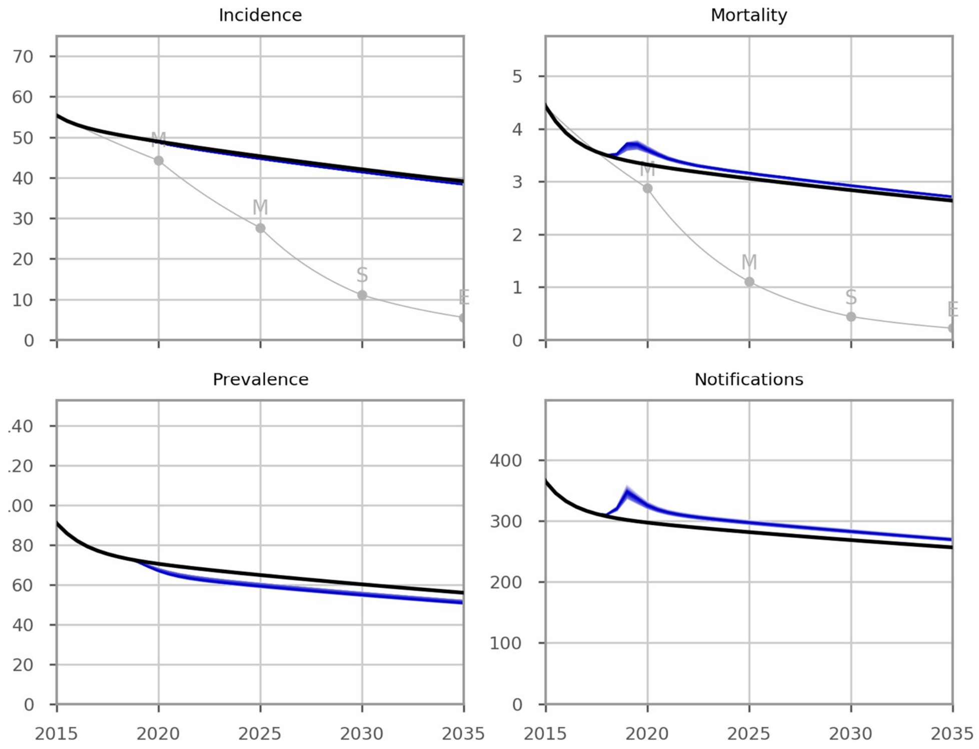

Figure S22. Effects of GeneXpert on TB epidemic.

Isoniazid preventive therapy (IPT)

Implementation

The WHO strongly recommends the systematic evaluation of contacts of potentially infectious cases of active TB as a form of intensified case finding for further cases of active disease. The investigation of close/household contacts of higher risk index cases (e.g. smear-positive pulmonary TB) is strongly recommended, while the investigation of all close/household contacts is conditionally recommended. Following such screening for active TB, preventive therapy is then recommended for contacts at the highest risk of progression to active disease following infection. However, the groups for which treatment is recommended has been highly restricted, focusing on PLHIV and children aged under five years [47]. Moreover, screening policies vary substantially between countries, and recommendations are inconsistently implemented [48]. At least seven meta-analyses have estimated the sensitivity of TST in diagnosing LTBI, with results ranging from 0.70 to 0.82 [49]. Meanwhile, meta-analytic estimates for the sensitivity of QuantiFERON range from 0.66 to 0.83, and of T-SPOT range from 0.62 to 0.90. Therefore, there is no evidence that any one of these tests has superior sensitivity to the others, and all the tests have a sensitivity of approximately 0.7 to 0.8.

A Cochrane review of IPT was undertaken in 1994, with the 2003 update to this review finding no further citations, such that the authors do not plan any further updates. The review was limited to randomised trials of appropriately dosed IPT provided for at least six months with follow-up for at least two years. It found IPT to be associated with a risk ratio for active TB of 0.40 (95% CI 0.31 to 0.52) [50]. This estimate is very similar to the results of a recent meta-analysis, which found a risk ratio of 0.41 (95% CI 0.19 to 0.80) [51]. When six-month and 12-month courses were considered separately, the respective risk ratios were 0.44 and 0.38, although this difference was not statistically significant. Rates of hepatotoxicity for these two regimen durations were 0.36% and 0.52%, respectively. For model implementation, it should be noted that all but one of the studies included in this review were analysed by intention-to-treat. Therefore, this estimate does not need to be reduced to reflect patient compliance.

Concerns regarding the relatively low efficacy of six-month isoniazid treatment and poor completion of twelve-month isoniazid treatment have resulted in a re-analysis of data from several clinical trials [52]. This re-analysis suggests that the optimal duration of isoniazid is nine months, and that this is associated with a 90% reduction in the risk of progression to active TB in fully compliant patients. Extending to 12 months is unlikely to improve efficacy any further. The findings of this re-analysis have resulted in the recommendations for nine months of INH by the American Thoracic Society in 2000, followed by health authorities of other countries soon after.

Recently, a randomised control trial (RCT) compared treatment efficacy of three-month weekly INH plus rifapentine and nine-month INH alone [53]. The combination therapy was found to be as effective as INH alone, but associated with less hepatotoxicity.

As this intervention is considered to target household contacts specifically, the first step in model implementation is to estimate the proportion of infections that occur at the household level; we estimate this at 60% by default, but encourage users to modify this value. Since only contacts of detected cases are detectable, this value is next multiplied by the proportion of active cases that are detected by the health system, which is modelled explicitly through the case detection rate. Last, this product is multiplied by the sensitivity of the diagnostic test employed for LTBI. All of the age and age group-specific IPT programs can each be implemented. If these programs are implemented together, the sum of the coverage of the two programs is used to determine the aggregate coverage for each age group that is specifically targeted. The resulting proportion of infections effectively targeted by IPT are subtracted from the force of infection and moved instead to IPT compartments, where patients remain for the duration of treatment (set by default to nine months through) before moving to the partially immune susceptible compartments applicable to their age group, treatment history, and risk group status.

Results

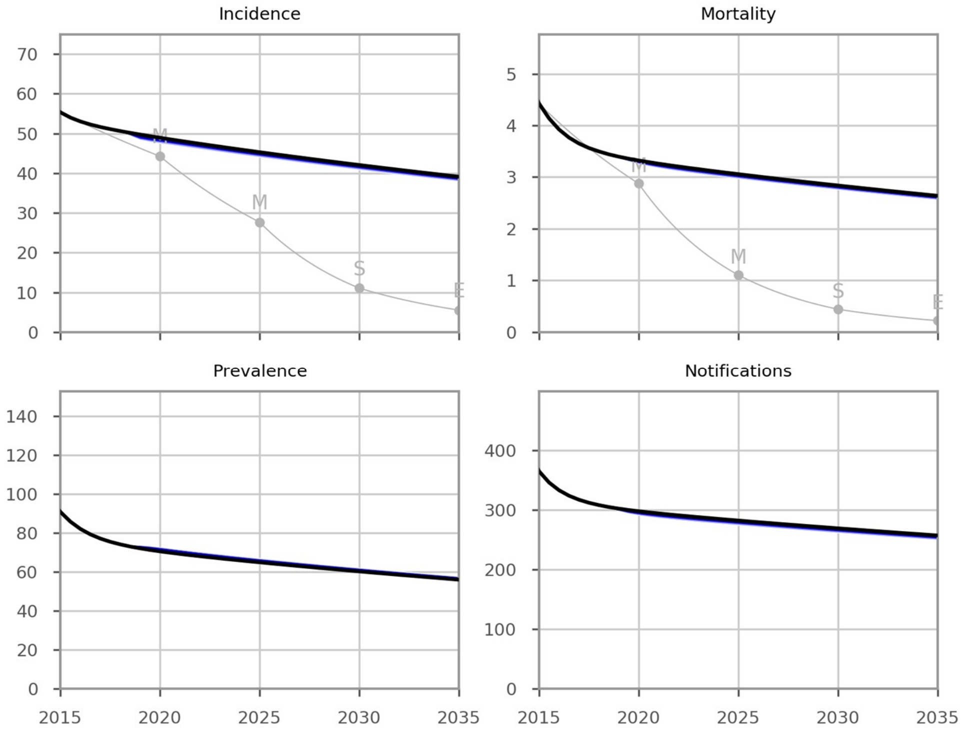

Figure S23. Effects of IPT on TB epidemic with intervention uncertainty.

Active case finding

Implementation

Rationale

To effectively decrease the transmission of *Mtb*, the duration of infectiousness should be kept to a minimum, although there are no specific targets for what an acceptable time to diagnosis is, nor is this time period required reporting for national TB control programs (NTPs) [54]. The delay to treatment initiation has been found to be significantly associated with the proportion of household contacts infected, with one study finding a delay of greater than 30 days to be an important threshold [55]. A systematic review found that a variety of features of the disease, patient, health system, culture, and environment may be important in influencing the time to presentation [56]. However, detecting patients early can be challenging, given that a small but significant proportion of patients report no symptoms at all, while a sizeable proportion (as many as half) do not report symptoms that are consistent with the commonly utilised criteria [57]. A substantial proportion of all cases of TB are still missed entirely [58], while both patient and health system-related delays to treatment initiation are substantial in a range of settings [59].

Historical background

Active case finding (ACF) through the chest radiography of asymptomatic persons was implemented extensively in developed country settings from the 1930s to the 1960s. Although this strategy was successful in detecting a significant number of previously undiagnosed cases, the financial expense and logistics were considerable. From the early 1960s, the paradigm began to change in the light of studies, finding that most patients with undiagnosed TB had symptoms for some time, and had often sought care previously. These studies suggested that few patients would be missed by restricting ACF to those with symptoms, such as having a cough for two weeks or more. Therefore, the focus shifted to health system strengthening with ACF considered unnecessary, particularly at a time when TB was thought to be becoming a relatively minor problem in global health. Since then, a range of ACF strategies have been studied, although few have been linked to prevalence surveys that could shed light on the proportion of undiagnosed cases detected [60].

DETECTB

One of the most important studies in ACF was DECECTB, which was a cluster randomised study of two alternative approaches to ACF undertaken in Harare from 2006 to 2008 [61]. This study compared six rounds of ACF at six-monthly intervals, either through the presence of a mobile van in the community for five days (manned by three lay workers equipped with a loudspeaker and leaflets) or through door-to-door enquiry at the household level (with each household approached once per ACF round by two teams of three lay workers). Two sputum specimens were collected from individuals with a history of cough for two weeks or longer and analysed with smear microscopy (but not culture). The primary outcome was the relative

yield of smear-positive TB between the two screening methods, while the secondary outcome was the change in culture-positive TB through prevalence surveys performed with the first and last ACF round.

Consistent with evidence from elsewhere (Thailand), the door-to-door approach did not lead to a marked increase in the number of cases diagnosed [62]. In fact, the mobile van intervention detected considerably more cases (255) than did the door-to-door intervention (137), for a cumulative yield of 4.27 and 2.38 smear-positive cases per 1000 population over the six rounds of intervention (or 0.71 and 0.40 per round). Given that the prevalence was 4.0 smear-positive cases per 1000 population at the start of the intervention and 2.3 smear-positive cases per 1000 per population before the fifth round and assuming a linear decrease in burden over the study period (as the authors did), the average prevalence of smear-positive disease over the course of the study would have been 3.1 per 1000. Therefore, the 0.71 smear-positive cases diagnosed in each round of van-based ACF and 0.40 cases in each round of door-to-door based ACF represent 23.2% and 12.8% of all the undiagnosed cases respectively.

Other recent studies

Sekandi et al. reported on a single round of ACF undertaken in a peri-urban setting of around 10,000 persons, finding 33 additional cases of smear-positive TB through an algorithm of symptom screening for chronic cough and smear microscopy [63]. This study was limited by comparison to DETECTB, in that no prevalence surveys were undertaken, such that the proportion of all undiagnosed cases that this represents is difficult to estimate. Given Uganda’s prevalence of TB of around 150 to 200 per 100,000 with around 60% of notified cases smear-positive, the smear-positive prevalence may be around 100 per 100,000. Therefore, it could be estimated that around 10 undiagnosed such cases should be present in a population of 10,000. Clearly, the study results refute this estimate, and indicate that the true prevalence was much higher in this slum setting, as the authors acknowledge.

A study of free workplace TB diagnostics included a prevalence survey at study completion, but not at the outset, and did not constitute active case finding in the sense reviewed here [64].

A comparison of cohorts of patients diagnosed by ACF and passive case finding in Cambodia found that the patients diagnosed through ACF were generally older (55 versus 48 years) and were considerably more likely to be smear-negative (71.4% versus 40.5%). Reassuringly for this intervention, treatment outcomes were comparable between the two groups, with treatment success rates of 96.4% and 95.2% for patients diagnosed through ACF and passive case finding, respectively [65]. Another study in the same country found that 810 cases of active TB were diagnosed through an ACF intervention, after the screening of 35,005 patients with chest x-ray and symptoms and testing of 3649 of these with GeneXpert. The intervention covered 30 operational districts with a population of approximately 100,000 to 200,000 each, and a TB incidence of around 176 cases per 100,000 per year [66,67]. The authors estimated that an additional 9.8% of cases had been found compared to the baseline

case detection rate, but if the expected decline in burden is allowed for, an additional 18.5% of cases were detected. As the total population in the intervention area was around 2.9 million, the proportion of the population screened was around 1.21%, and the proportion of the population definitively tested was 0.126%.

Conclusions and model implementation

For smear-based ACF, we estimate that van-based ACF implemented with screening rounds at six-monthly intervals can be expected to diagnose around 23.2% of smear-positive cases not previously diagnosed at each round. For GeneXpert-based strategies, the intervention can also be reasonably applied to the undiagnosed smear-negative population, but is multiplied by the sensitivity of GeneXpert for smear-negative disease (i.e. 67%, to give an overall proportion diagnosed of 15.6%). As these strategies generally commence with enquiry as to the presence of cough, no effect on extrapulmonary disease is expected. In this analysis, we consider the intervention to be directed at the general community.

Results

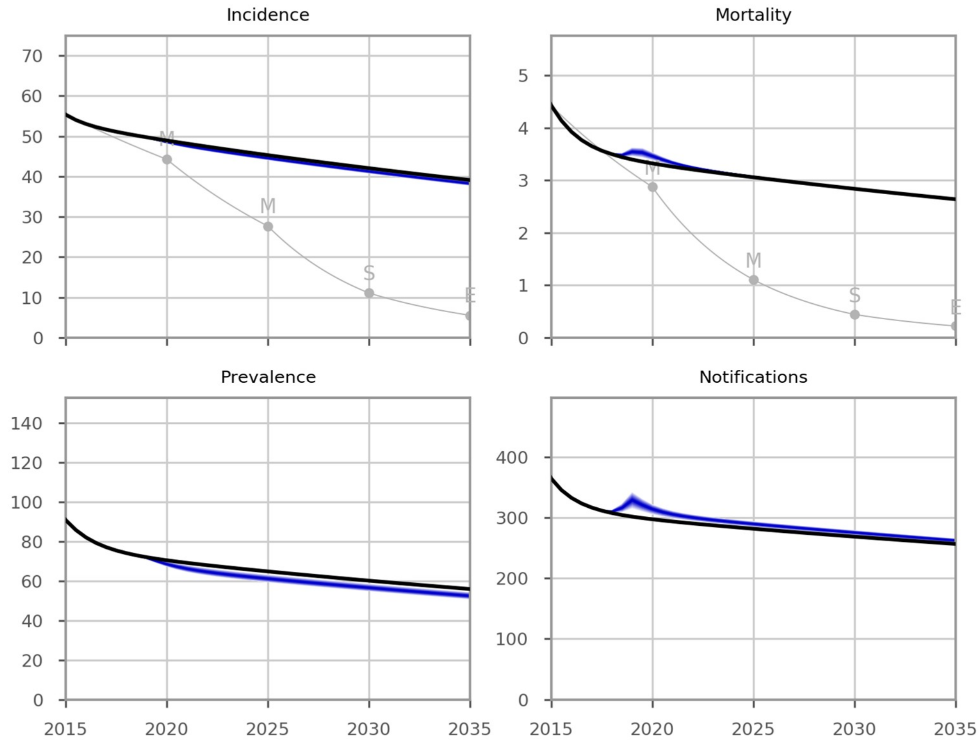

Figure S24. Effects of GeneXpert-based ACF on TB epidemic with intervention uncertainty.

Awareness raising

Implementation

Background

Cultural, environmental, and politico-economic factors are known to be important in driving the TB epidemic, which is more often conceptualised from a biological perspective [68]. Therefore, communication interventions for TB control have the potential to markedly improve rates of presentation for care through reduction in stigma, improved understanding of the condition, and greater community awareness. Countries such as Peru and Vietnam have used improved awareness as a cornerstone of their control efforts [69–71]. Although a systematic review was registered in 2014 to consider the effect of mass media interventions on TB knowledge, attitudes, awareness, health care seeking behaviour, and health service utilisation, no results are yet available from this study. Moreover, there are a number of critical considerations to bear in mind in estimating the likely effectiveness of such interventions, including tailoring messages to the context and culture in which they are provided, and ensuring that curative care of a sufficient standard is available to the additional persons who may present for care as a result of the intervention.

TB-specific studies

Studies investigating the impact of awareness programs on TB have reported variable results, but have consistently found increased rates of presentation for care, increased detection of new active and latent cases, and reduced rates of treatment abandonment [72]. In a study conducted in an indigenous community with high TB burden in Canada in the context of passive case finding, Alvarez et al. found that the number of people presenting themselves to clinics increased by 92% during the four-month community-wide awareness campaigns, compared to the previous periods without the campaign (increased from an average of 26 people per month to 50 per month) [73]. This study also found that a door-to-door campaign increased the number of new LTBI cases by 34%, and increased the number of newly diagnosed active TB cases by 29.5%. In Columbia, Jaramillo reported that a six-week mass media-based health education campaign (radio, television, and newspaper) increased the number of smears processed by laboratories by 64%, and increased the number of smear-positive pulmonary TB cases detected and notified by 52% [74]. However, this increase only accrued in the study area and for the quarter during which the intervention was implemented, with programmatic effects not sustained after the intervention for either study.

Awareness programs have also been found to enhance treatment retention. A report using nationwide data in Peru found that health communication steadily reduced the rate of TB treatment abandonment from 12.1% prior to the start of the program to 2.8% over a period of 10 years. A brief report of an intervention in Odisha (India) involving health worker education, van-mounted loudspeakers, and community-based health camps found an increase in the number of detections [75]. Although the increase in the total number of TB diagnoses made was not reported, the number of persons screened increased by 87.8% and the number

of smear-positive diagnoses made increased by 10.8% (figures that seem consistent with those reported by the studies discussed above).

Conclusions and model implementation

Implementation of this intervention acts to accelerate the rate of case detection from baseline, and similarly the rate at which missed cases accrue through incorrect diagnosis, reflecting a decrease in pre-presentation delays to treatment. The applied value for the relative increase in presentation rate is 1.52-fold the baseline rate, using the results of the study by Jaramillo et al. above. This is intended to reflect intensive, periodic advertising and awareness raising through the mass media, but without the extensive community mobilisation described in Alvarez et al. above. An even more extensive intervention could be captured by implementing a relative increase parameter of 1.92 or similar. However, we believe that the value of 1.52 better reflects the interventions considered in Fiji.

An important caveat is that we aim to simulate repeated media-based interventions in order to maintain the effectiveness of this intervention over time, assuming interventions of six weeks in duration implemented quarterly or similar. We also assume that the effect of the intervention is maintained over time, ignoring the potential for loss of effectiveness with time, which is impossible to quantify.

Results

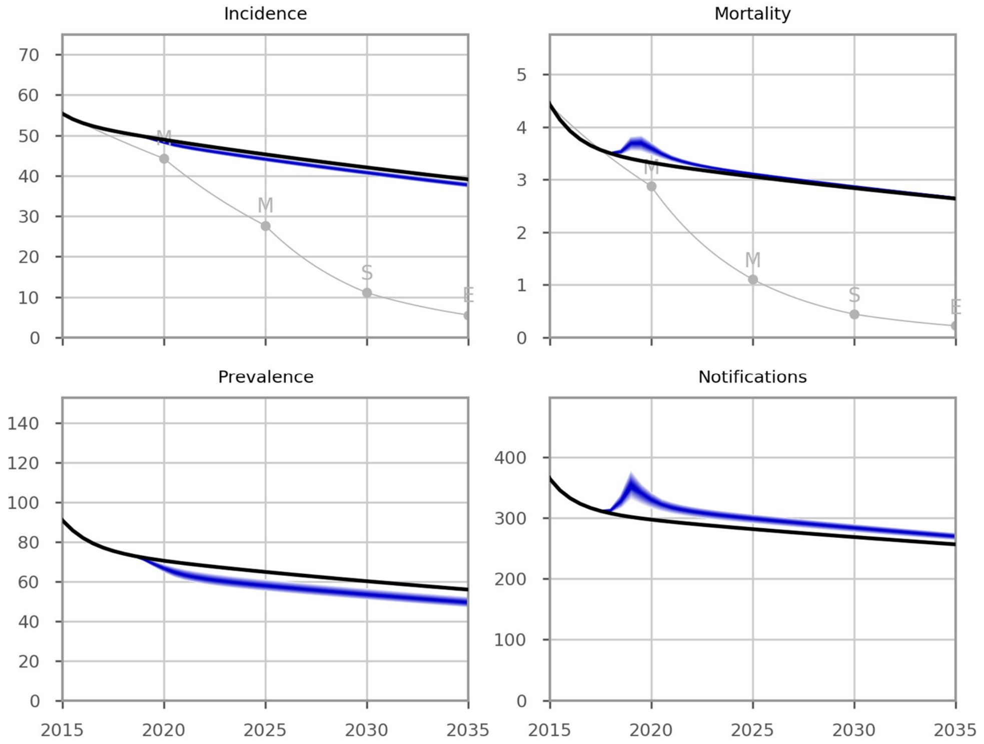

Figure S25. Effects of awareness raising on TB epidemic with intervention uncertainty.

Effect of interventions on actual mortality

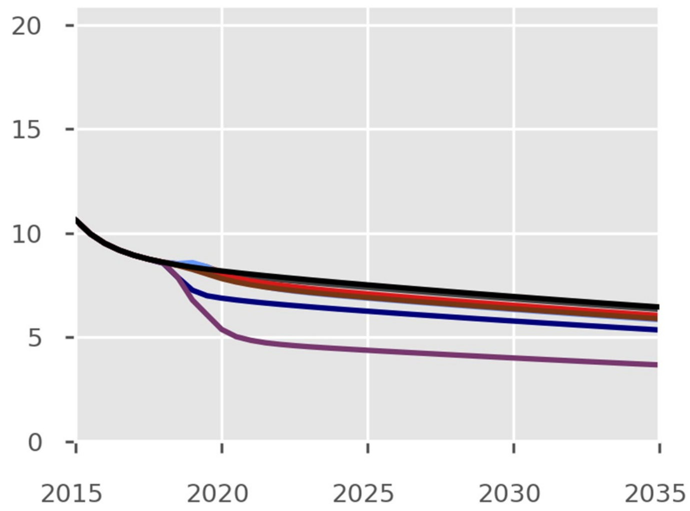

Figure S26. Effects of interventions on true mortality. This includes all the modelled mortality occurring outside of the health system.

Cost inputs for economic analysis

Support for patients under treatment

In Fiji, this intervention involves getting a person trained in directly observed therapy short course (DOTS) to administer daily treatment and being given USD 10 per day while they are a DOTS trained-treatment administering person. The Fiji NTP estimates that the total cost of the program in 2015 is about USD 150,000 with a total annual number of patients to be treated of 305, giving a unit program cost of USD 500 (USD 534 in 2017 values). The yearly cost of treatment support is projected by the NTP to be USD 3469 for the next three years (USD 10,407 for a total of three years) to scale up the program; however, cost components of this amount are not available.

Decentralisation

Fiji-specific cost data for the decentralisation of TB care are lacking, nor are such data available in the literature. In consultation with the Fiji NTP, we assumed a unit cost of USD 1000 per patient for decentralization. An arbitrary value of USD 700,000 for start-up cost for three years was used.

GeneXpert

Using an ingredient costing approach of identifying all the inputs and quantities required to perform the test such as cartridge, equipment, and salaries, Vassall et al. [81] estimated that the cost for the GeneXpert test ranged from USD 23 in India to USD 39 in South Africa and USD 47 in Uganda. These estimates are consistent with those from Dowdy et al. [82] and Menzies et al. [83]. Although the WHO recommends the rapid implementation of GeneXpert because of its proven benefits for the diagnosis of TB, implementation among resource-constrained countries has been slow due to the substantial start-up costs of GeneXpert, which is estimated to be $103,679.5 for Fiji per year for three years. This estimate includes the following cost components:

- WHO to organise technical assistance (TA) to strengthen NTP to improve diagnosis using new technology (GeneXpert), culture and DST–QMRL (USD 19,114.46)
- NTP to organise a workshop on improving diagnosis using GeneXpert, culture, and DST by Technical Assistant (USD 85.28)
- NTP to organise training on GeneXpert and a portable X-ray machine for staff with assistance of the WHO TB and Leprosy MO (USD 426.41)
- GeneXpert kits: Xpert^{R}MTB/RIF, GXMTB/RIF-10 tests/kit, Cepheid; Gene Xpert Machine (USD 36,961)
- Maintenance cost for equipment: GeneXpert calibration kits, GeneXpert replacement module, and GeneXpert warranty extension (USD 27,312)
- Recruitment and appointment of TA to strengthen NTP to improve diagnosis using new technology (GeneXpert), culture, and DST–QMRL under WHO implementation for one visit per annum (USD 12,183.98)
- TA to conduct workshop and training on improving diagnosis using new technology (GeneXpert), culture and DST–QMRL (USD 1745.53)

- Conduct training and retraining on GeneXpert and intensified case finding (portable X-ray screening) for laboratory technicians and TB medical officers and TB nurse coordinators in collaboration with the WHO TB and Leprosy MO (USD 2230)
- Procure and distribute consumables for GeneXpert (USD 3620)

Isoniazid preventive therapy

As with most TB programs in Fiji, country-specific cost data for IPT are not available. As such, we performed a literature search to inform the cost inputs for the program. Although a few studies that investigated the economics of six-month IPT exist, most of these studies focused on IPT in HIV-infected adult patients [76–79]. To date, there is only one study that investigated the cost and cost-effectiveness of IPT in children contacts (<5 years of age), which are the target population group of the IPT program in Fiji [80]. Mandalakas et al. [80] estimated that the unit costs of IPT in this setting were USD 179 for health providers. These estimates included costs of screening using TST, medications, clinic visits, and liver function tests. In Fiji, the current coverage of IPT in children under five years old is only 23.6%, and the country aspires to scale up the program to achieve a high level of coverage of 80% in the next few years. Sutton et al. [78] estimated the cost required for starting an IPT program to be USD 105.35 per patient, which includes costs of personnel, laboratory use, supplies, capital, and infrastructure. Under most simulations, our model projected that the total number of children over the next three years who are eligible for the program is around 672, such that the total start-up cost was estimated to be USD 70,800.

Active case finding

Evidence on the cost of active case finding is limited. The total per patient cost is estimated to be USD 74 in urban Africa from a health provider perspective [63]. These costs include program costs (administration, transportation, communication, health personnel), direct medical costs (smear tests, culture tests, chest X-ray, consumable supplies), and patient and caregiver costs (for analysis from societal perspective). Of note, sputum smear microscopy is used for TB screening and diagnosis in this study. Then, the unit cost of GeneXpert-based ACF is calculated as follows: unit cost of GeneXpert-based ACF = unit cost of smear-based ACF (USD 74.26) – unit cost of smear test (USD 1.16) + unit cost of GeneXpert test (USD 22.63) = USD 95.73

In Fiji, the activities and their estimated costs that are related to the implementation of active case finding program are identified follows:

- Mobile caravan for active case finding in communities (USD 116,402.68)
- Six-monthly supervisory visits to hard-to-reach populations (USD 1384.95)
- Weekly and quarterly review of surveillance data to identify high-risk groups (USD 2544.23)
- Active screening of TB symptoms among contacts in hard-to-reach areas (USD 11,811.78)

- Six-monthly outreach clinics to 20 medical areas and hard-to-reach populations (USD 1491.85)
- Monthly TB clinics in HIV hubs for active case finding and the provision of IPT for PLHIV (USD 384.84)
- Repairs and maintenance for mobile caravan (USD 9312.21)
- Fuel and oil for mobile caravan (USD 9407.72)
- Training and retraining on GeneXpert and intensified case finding (screening by portable X-ray) for laboratory technicians and TB medical officers and TB nurse coordinators in collaboration with the WHO TB and Leprosy MO (USD 2230.79)
- Active screening of high-risk groups in the 20 medical areas and hard-to-reach populations (USD 766)
- Mobile caravan for active case finding outreach activities for a portable X-ray machine and GeneXpert machine (USD 9465.46)
- Technical assistance from WHO to strengthen the NTP to improve diagnosis using new technology (GeneXpert), culture, and DST–QMRL (USD 19,114.46)
- Workshop on improving diagnosis using GeneXpert, culture, and DST for technical assistant (USD 85.28)
- Training on GeneXpert and the portable X-ray machine for staff with assistance of the WHO TB and Leprosy MO (USD 426.41)
- GeneXpert kits, GeneXpert machine (USD 36,961)
- Maintenance cost for equipment, GeneXpert calibration kits, GeneXpert replacement module, GeneXpert warranty extension (USD 27,312)
- Recruitment and appointment of technical assistants to strengthen the NTP to improve diagnosis using new technology (GeneXpert), culture, and DST–QMRL under WHO implementation for one visit per annum (USD 12,183.98)
- Workshop and training on improving diagnosis using new technology (GeneXpert), culture, and DST–QMRL (USD 1745.53)
- Consumables for GeneXpert (USD 3620)

Based on the above activities and costs, the yearly start-up costs for GeneXpert-based active case finding are estimated to be USD 266,651. We assume that the start-up cost is applied for three years, with the cost of purchasing mobile caravans (USD 116,402.68) excluded from year 2 and year 3.

Awareness raising

No economic or other data were available to inform the estimated costs of this intervention, and an arbitrary cost was arrived at in consultation with local programmatic staff. Therefore, the economics of this intervention remain highly uncertain, and should be interpreted with caution.

Economic inputs

Intervention	***Start-up costs (USD)***	***Start-up duration (years)***	***Unit cost (USD)***	***Saturation (%)***
1. Treatment support	10,407	3	534	100
2. Decentralisation	700,000	3	1,000	80
3. GeneXpert	311,039	3	23	100
4. IPT	70,800	3	179	80
5. ACF	567,148	3	95.73	90
6. Awareness	0	0	10	90

Table S2. Economic input values.

Cost coverage curves

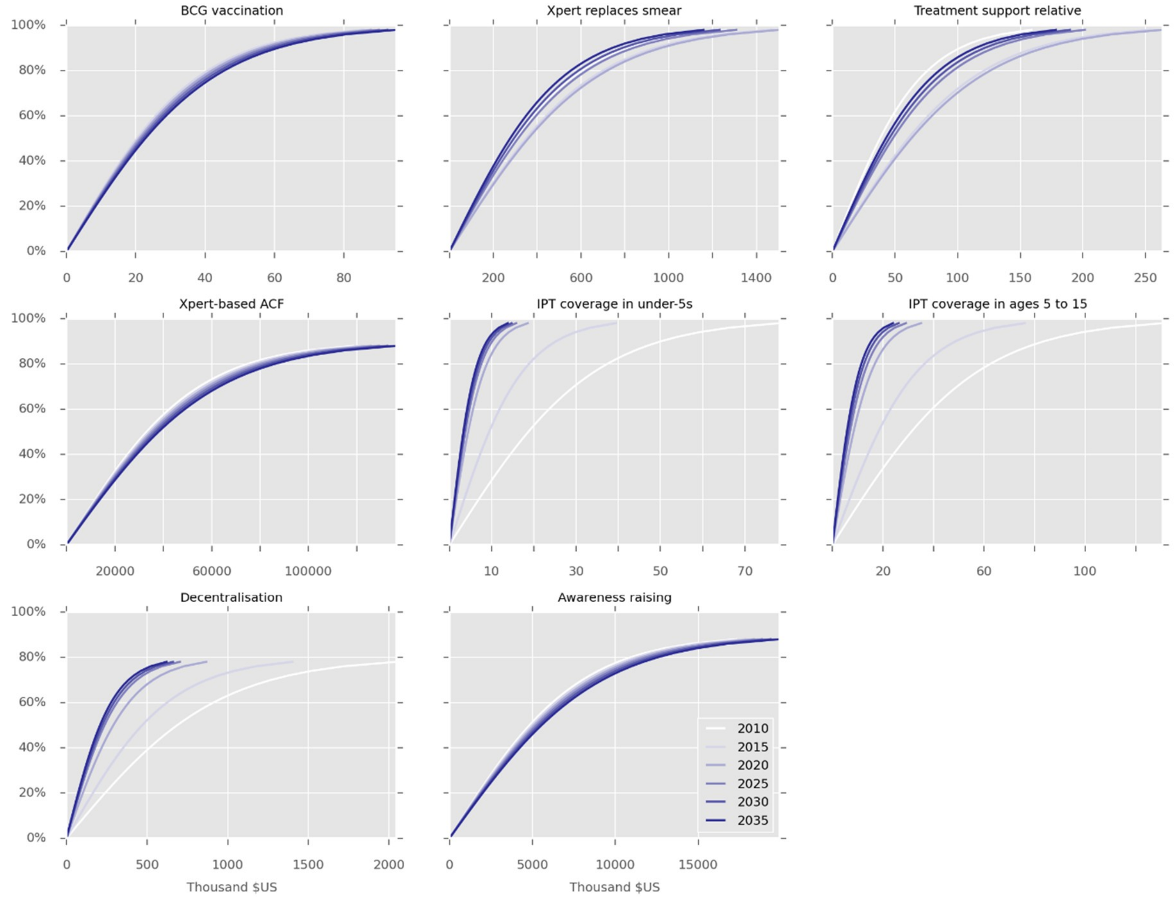

Figure S27. Cost-coverage curves under Scenario 7 for all the interventions combined. Note that these are presented as an example because each intervention affects the cost coverage of each other intervention. Therefore, different cost-coverage curves apply under each scenario.

References

1. Trauer, J. M., Ragonnet, R., Doan, T. N. & McBryde, E. S. Modular programming for tuberculosis control, the "AuTuMN" platform. *BMC Infect. Dis.* **17,** 546 (2017).
2. The World Bank. World Development Indicators | DataBank.
3. World Bank. Life expectancy at birth, total (years) | Data. Available at: http://data.worldbank.org/indicator/SP.DYN.LE00.IN. (Accessed: 19th June 2017)
4. Andrews, J. R. *et al.* Risk of progression to active tuberculosis following reinfection with Mycobacterium tuberculosis. *Clin Infect Dis* **54,** 784–791 (2012).
5. Verver, S. *et al.* Rate of reinfection tuberculosis after successful treatment is higher than rate of new tuberculosis. *Am J Respir Crit Care Med* **171,** 1430–1435 (2005).
6. Gomes, M. G. *et al.* How host heterogeneity governs tuberculosis reinfection? *Proc Biol Sci* **279,** 2473–2478 (2012).
7. Trauer, J. M. *et al.* Risk of active tuberculosis in the five years following infection ⋯ 15%? *Chest* **149,** (2016).
8. Sloot, R., Schim van der Loeff, M. F., Kouw, P. M. & Borgdorff, M. W. Risk of Tuberculosis after Recent Exposure. A 10-Year Follow-up Study of Contacts in Amsterdam. *Am J Respir Crit Care Med* **190,** 1044–1052 (2014).
9. Ragonnet, R. *et al.* Optimally capturing latency dynamics in models of tuberculosis transmission. *Epidemics* **21,** 39–47 (2017).
10. Dowdy, D. W., Dye, C. & Cohen, T. Data Needs for Evidence-Based Decisions: a Tuberculosis Modeler's 'Wish List'. *Int J Tuberc Lung Dis* **17,** 866–877 (2013).
11. Tiemersma, E. W., van der Werf, M. J., Borgdorff, M. W., Williams, B. G. & Nagelkerke, N. J. Natural history of tuberculosis: duration and fatality of untreated pulmonary tuberculosis in HIV negative patients: a systematic review. *PLoS One* **6,** e17601 (2011).
12. World Health Organization. Global Tuberculosis Report 2016. **2016,** (2016).
13. Marais, B. J. *et al.* The natural history of childhood intra-thoracic tuberculosis: a critical review of literature from the pre-chemotherapy era. *Int J Tuberc Lung Dis* **8,** 392–402 (2004).
14. Cruz, A. T. & Starke, J. R. Clinical manifestations of tuberculosis in children. *Paediatr. Respir. Rev.* **8,** 107–117 (2007).
15. Matlow, A., Robb, M. & Goldman, C. Infection control and paediatric tuberculosis: A practical guide for the practicing paediatrician. *Paediatr. Child Health* **8,** 624–6 (2003).
16. Starke, J. R. Transmission of mycobacterium tuberculosis to and from children and adolescents. *Semin. Pediatr. Infect. Dis.* **12,** 115–123 (2001).
17. Hussey, G., Chisholm, T. & Kibel, M. Miliary tuberculosis in children: a review of 94 cases. *Pediatr. Infect. Dis. J.* **10,** 832–6 (1991).
18. Straetemans, M., Glaziou, P., Bierrenbach, A. L., Sismanidis, C. & van der Werf, M. J. Assessing tuberculosis case fatality ratio: a meta-analysis. *PLoS One* **6,** e20755 (2011).

19. Pablos-Méndez, A., Blustein, J. & Knirsch, C. A. The role of diabetes mellitus in the higher prevalence of tuberculosis among Hispanics. *Am. J. Public Health* **87,** 574–9 (1997).
20. Alisjahbana, B. *et al.* Diabetes mellitus is strongly associated with tuberculosis in Indonesia. *Int. J. Tuberc. Lung Dis.* **10,** 696–700 (2006).
21. Kim, S. J., Hong, Y. P., Lew, W. J., Yang, S. C. & Lee, E. G. Incidence of pulmonary tuberculosis among diabetics. *Tuber. Lung Dis.* **76,** 529–33 (1995).
22. Ponce-De-Leon, A. *et al.* Tuberculosis and diabetes in southern Mexico. *Diabetes Care* **27,** 1584–90 (2004).
23. Pérez, A., Brown, H. S., Restrepo, B. I. & RESTREPO, B. I. Association between tuberculosis and diabetes in the Mexican border and non-border regions of Texas. *Am. J. Trop. Med. Hyg.* **74,** 604–11 (2006).
24. Oluboyo, P. O. & Erasmus, R. T. The significance of glucose intolerance in pulmonary tuberculosis. *Tubercle* **71,** 135–8 (1990).
25. Başoğlu, O. K., Bacakoğlu, F., Cok, G., Sayiner, A. & Ateş, M. The oral glucose tolerance test in patients with respiratory infections. *Monaldi Arch. chest Dis. = Arch. Monaldi per le Mal. del torace* **54,** 307–10 (1999).
26. Jeon, C. Y. & Murray, M. B. Diabetes mellitus increases the risk of active tuberculosis: a systematic review of 13 observational studies. *PLoS Med* **5,** e152 (2008).
27. Thiam, S. *et al.* Effectiveness of a Strategy to Improve Adherence to Tuberculosis Treatment in a Resource-Poor Setting. *JAMA* **297,** 380 (2007).
28. Davtyan, K. *et al.* Performance of decentralised facilities in tuberculosis case notification and treatment success in Armenia. *Public Heal. action* **4,** S13-6 (2014).
29. Shaweno, D., Shaweno, T., Trauer, J. M., Denholm, J. T. & McBryde, E. S. Heterogeneity of distribution of tuberculosis in Sheka Zone, Ethiopia: Drivers and temporal trends. *Int. J. Tuberc. Lung Dis.* **21,** (2017).
30. Ketema, K. H., Raya, J., Workineh, T., Klinkenberg, E. & Enquselassie, F. Does decentralisation of tuberculosis care influence treatment outcomes? The case of Oromia Region, Ethiopia. *Public Heal. action* **4,** S13-7 (2014).
31. Egwaga, S. *et al.* Patient-centred tuberculosis treatment delivery under programmatic conditions in Tanzania: a cohort study. *BMC Med.* **7,** 80 (2009).
32. World Health Organization. *Framework for Implementing New Tuberculosis Diagnostics*. (2010).
33. WHO endorses new rapid tuberculosis test, a major milestone for global TB diagnosis and care. *WHO* (2010). Available at: http://www.who.int/mediacentre/news/releases/2010/tb_test_20101208/en/. (Accessed: 21st August 2017)
34. Stevens, W. S., Scott, L., Noble, L., Gous, N. & Dheda, K. Impact of the GeneXpert MTB/RIF Technology on Tuberculosis Control. *Microbiol. Spectr.* **5,** (2017).
35. Steingart, K. R. *et al.* Xpert(R) MTB/RIF assay for pulmonary tuberculosis and

rifampicin resistance in adults. *Cochrane Database Syst Rev* **1,** CD009593 (2013).

36. Boehme, C. C. *et al.* Feasibility, diagnostic accuracy, and effectiveness of decentralised use of the Xpert MTB/RIF test for diagnosis of tuberculosis and multidrug resistance: a multicentre implementation study. *Lancet* **377,** 1495–1505 (2011).
37. Hanrahan, C. F. *et al.* Time to Treatment and Patient Outcomes among TB Suspects Screened by a Single Point-of-Care Xpert MTB/RIF at a Primary Care Clinic in Johannesburg, South Africa. *PLoS One* **8,** e65421 (2013).
38. Omrani, A. S. *et al.* GeneXpert MTB/RIF Testing in the Management of Patients with Active Tuberculosis; A Real Life Experience from Saudi Arabia. *Infect. Chemother.* **46,** 30–4 (2014).
39. Stagg, H. R. *et al.* Decreased Time to Treatment Initiation for Multidrug-Resistant Tuberculosis Patients after Use of Xpert MTB/RIF Test, Latvia. *Emerg. Infect. Dis.* **22,** 482–90 (2016).
40. Naidoo, P. *et al.* A Comparison of Multidrug-Resistant Tuberculosis Treatment Commencement Times in MDRTBPlus Line Probe Assay and Xpert® MTB/RIF-Based Algorithms in a Routine Operational Setting in Cape Town. *PLoS One* **9,** e103328 (2014).
41. Steingart, K. R. *et al.* Fluorescence versus conventional sputum smear microscopy for tuberculosis: a systematic review. *Lancet Infect Dis* **6,** 570–581 (2006).
42. Theron, G. *et al.* Do high rates of empirical treatment undermine the potential effect of new diagnostic tests for tuberculosis in high-burden settings? *Lancet Infect Dis* **14,** 527–532 (2014).
43. Walusimbi, S. *et al.* Meta-analysis to compare the accuracy of GeneXpert, MODS and the WHO 2007 algorithm for diagnosis of smear-negative pulmonary tuberculosis. *BMC Infect. Dis.* **13,** 507 (2013).
44. Chang, K. C., Leung, C. C., Yew, W. W. & Tam, C. M. Supervised and induced sputum among patients with smear-negative pulmonary tuberculosis. *Eur. Respir. J.* **31,** 1085–1090 (2008).
45. Accuracy and Feasibility of Xpert Ultra, ClinicalTrials.gov. Available at: https://clinicaltrials.gov/ct2/show/NCT03072576. (Accessed: 21st August 2017)
46. Cepheid. 2017 launch of new TB test Ultra backed by WHO recommendation. Available at: http://www.cepheid.com/us/about-us/news-events/press-releases/216-2017-launch-of-new-tb-test-ultra-backed-by-who-recommendation. (Accessed: 21st August 2017)
47. World Health Organization. *Recommendations for investigating contacts of persons with infectious tuberculosis in low- and middle-income countries*. *WHO* (World Health Organization, 2012).
48. Hwang, T. J., Ottmani, S. & Uplekar, M. A rapid assessment of prevailing policies on tuberculosis contact investigation. *Int. J. Tuberc. Lung Dis.* **15,** 1620–1623 (2011).
49. Sollai, S., Galli, L., de Martino, M. & Chiappini, E. Systematic review and meta-

analysis on the utility of Interferon-gamma release assays for the diagnosis of Mycobacterium tuberculosis infection in children: a 2013 update. *BMC Infect. Dis.* **14,** S6 (2014).

50. Smieja, M. J., Marchetti, C. A., Cook, D. J. & Smaill, F. M. Isoniazid for preventing tuberculosis in non-HIV infected persons. *Cochrane Database Syst Rev* CD001363 (2000). doi:10.1002/14651858.CD001363
51. Pease, C. *et al.* Efficacy and completion rates of rifapentine and isoniazid (3HP) compared to other treatment regimens for latent tuberculosis infection: a systematic review with network meta-analyses. *BMC Infect. Dis.* **17,** 265 (2017).
52. Comstock, G. W. How much isoniazid is needed for prevention of tuberculosis among immunocompetent adults? *Int. J. Tuberc. Lung Dis.* **3,** 847–50 (1999).
53. Sterling, T. R. *et al.* Three months of rifapentine and isoniazid for latent tuberculosis infection. *N Engl J Med* **365,** 2155–2166 (2011).
54. Lonnroth, K. *et al.* Tuberculosis control and elimination 2010-50: cure, care, and social development. *Lancet* **375,** 1814–1829 (2010).
55. Lin, X., Chongsuvivatwong, V., Lin, L., Geater, A. & Lijuan, R. Dose–response relationship between treatment delay of smear-positive tuberculosis patients and intra-household transmission: a cross-sectional study. *Trans. R. Soc. Trop. Med. Hyg.* **102,** 797–804 (2008).
56. Storla, D. G., Yimer, S. & Bjune, G. A. A systematic review of delay in the diagnosis and treatment of tuberculosis. *BMC Public Health* **8,** 15 (2008).
57. Ayles, H. *et al.* Prevalence of Tuberculosis, HIV and Respiratory Symptoms in Two Zambian Communities: Implications for Tuberculosis Control in the Era of HIV. *PLoS One* **4,** e5602 (2009).
58. World Health Organization, W. H. *Global tuberculosis report 2016. WHO* (2016). doi:ISBN 978 92 4 156539 4
59. Sreeramareddy, C. T., Panduru, K. V, Menten, J. & Van den Ende, J. Time delays in diagnosis of pulmonary tuberculosis: a systematic review of literature. *BMC Infect. Dis.* **9,** 91 (2009).
60. Golub, J. E., Mohan, C. I., Comstock, G. W. & Chaisson, R. E. Active case finding of tuberculosis: historical perspective and future prospects. *Int J Tuberc Lung Dis* **9,** 1183–1203 (2005).
61. Corbett, E. L. *et al.* Comparison of two active case-finding strategies for community-based diagnosis of symptomatic smear-positive tuberculosis and control of infectious tuberculosis in Harare, Zimbabwe (DETECTB): a cluster-randomised trial. *Lancet* **376,** 1244–1253 (2010).
62. Elink Schuurman, M. W. *et al.* The rapid village survey in tuberculosis control. *Tuber. Lung Dis.* **77,** 549–54 (1996).
63. Sekandi, J. N., Neuhauser, D., Smyth, K. & Whalen, C. C. Active case finding of undetected tuberculosis among chronic coughers in a slum setting in Kampala, Uganda. *Int J Tuberc Lung Dis* **13,** 508–513 (2009).

64. Corbett, E. L. *et al.* Epidemiology of tuberculosis in a high HIV prevalence population provided with enhanced diagnosis of symptomatic disease. *PLoS Med* **4,** e22 (2007).
65. Eang, M. T. *et al.* Early detection of tuberculosis through community-based active case finding in Cambodia. *BMC Public Health* **12,** 469 (2012).
66. Yadav, R. P., Satha, P., Nishikiori, N., Eang, M. T. & Lubell, Y. Cost-Effectiveness of a Tuberculosis Active Case Finding Program Targeting Household and Neighborhood Contacts in Cambodia. *Am. J. Trop. Med. Hyg.* **90,** 866–872 (2014).
67. Morishita, F. *et al.* Increased Case Notification through Active Case Finding of Tuberculosis among Household and Neighbourhood Contacts in Cambodia. *PLoS One* **11,** e0150405 (2016).
68. Ho, M.-J. Sociocultural aspects of tuberculosis: a literature review and a case study of immigrant tuberculosis. *Soc. Sci. Med.* **59,** 753–762 (2004).
69. Fernandez Llanos-Zavalaga, Patricia Poppe, Youssef Tawfik & Cathleen Church-Balin. *The Role of Communication in Peru's Fight Against Tuberculosis*. (2004).
70. Huu Thuy, D., Llanos-Zavalaga, F., Nguyen Thi Mai Huong, Poppe, P. & Youssef Tawfik. *The Role of Health Communication in Achieving Global TB Control Targets. Marie Stopes International Vietnam* (2004).
71. Thuy, D. H., Huong, N. T. M., Tawfik, Y. & Church-Balin, C. *The Role of Health Communication in Vietnam's Fight Against Tuberculosis*. (2004).
72. Nglazi, M. D. *et al.* The impact of mass media interventions on tuberculosis awareness, health-seeking behaviour and health service utilisation: a systematic review protocol. *BMJ Open* **4,** e004302 (2014).
73. Alvarez, G. G. *et al.* Taima (stop) TB: the impact of a multifaceted TB awareness and door-to-door campaign in residential areas of high risk for TB in Iqaluit, Nunavut. *PLoS One* **9,** e100975 (2014).
74. Jaramillo, E. The impact of media-based health education on tuberculosis diagnosis in Cali, Colombia. *Health Policy Plan.* **16,** 68–73 (2001).
75. Parija, D. *et al.* Impact of awareness drives and community-based active tuberculosis case finding in Odisha, India. *Int. J. Tuberc. Lung Dis.* **18,** 1105–1107 (2014).
76. Kapoor, S., Gupta, A. & Shah, M. Cost-effectiveness of isoniazid preventive therapy for HIV-infected pregnant women in India. *Int. J. Tuberc. Lung Dis.* **20,** 85–92 (2016).
77. Pho, M. T. *et al.* The cost-effectiveness of tuberculosis preventive therapy for HIV-infected individuals in southern India: a trial-based analysis. *PLoS One* **7,** e36001 (2012).
78. Sutton, B. S., Arias, M. S., Chheng, P., Eang, M. T. & Kimerling, M. E. The cost of intensified case finding and isoniazid preventive therapy for HIV-infected patients in Battambang, Cambodia. *Int. J. Tuberc. Lung Dis.* **13,** 713–8 (2009).
79. Maheswaran, H. & Barton, P. Intensive case finding and isoniazid preventative therapy in HIV infected individuals in Africa: economic model and value of information analysis. *PLoS One* **7,** e30457 (2012).
80. Mandalakas, A. M. *et al.* Modelling the cost-effectiveness of strategies to prevent

tuberculosis in child contacts in a high-burden setting. *Thorax* **68,** 247–55 (2013).

81. Vassall, A. *et al.* Rapid Diagnosis of Tuberculosis with the Xpert MTB/RIF Assay in High Burden Countries: A Cost-Effectiveness Analysis. *PLoS Med.* **8,** e1001120 (2011).

82. Dowdy, D. W., van't Hoog, A., Shah, M. & Cobelens, F. Cost-effectiveness of rapid susceptibility testing against second-line drugs for tuberculosis. *Int. J. Tuberc. Lung Dis.* **18,** 647–54 (2014).

83. Menzies, N. A., Cohen, T., Lin, H.-H., Murray, M. & Salomon, J. A. Population health impact and cost-effectiveness of tuberculosis diagnosis with Xpert MTB/RIF: a dynamic simulation and economic evaluation. *PLoS Med.* **9,** e1001347 (2012).
